# Synthesis and biological evaluation of 2-arylbenzofuran derivatives as potential anti-Alzheimer’s disease agents

**DOI:** 10.1080/14756366.2021.1940993

**Published:** 2021-06-16

**Authors:** Yinling Yun, Yuhang Miao, Xiaoya Sun, Jie Sun, Xiaojing Wang

**Affiliations:** Institute of Materia Medica, Shandong First Medical University and Shandong Academy of Medical Sciences, Jinan, China

**Keywords:** 2-Arylbenzofuran derivatives, Alzheimer's disease, cholinesterase inhibitors, β-secretase inhibitory activity

## Abstract

Alzheimer's disease (AD) is a type of progressive dementia caused by degeneration of the nervous system. A single target drug usually does not work well. Therefore, multi-target drugs are designed and developed so that one drug can specifically bind to multiple targets to ensure clinical effectiveness and reduce toxicity. We synthesised a series of 2-arylbenzofuran derivatives and evaluated their in vitro activities. 2-Arylbenzofuran compounds have good dual cholinesterase inhibitory activity and β-secretase inhibitory activity. The IC_50_ value of compound 20 against acetylcholinesterase inhibition (0.086 ± 0.01 µmol·L^−1^) is similar to donepezil (0.085 ± 0.01 µmol·L^−1^) and is better than baicalein (0.404 ± 0.04 µmol·L^−1^). And most of the compounds have good BACE1 inhibitory activity, of which 3 compounds (8, 19 and 20) show better activity than baicalein (0.087 ± 0.03 µmol·L^−1^). According to experimental results, 2-arylbenzofuran compounds provide an idea for drug design to develop prevention and treatment for AD.

## 1. Introduction

Alzheimer's disease (AD) is a type of progressive dementia caused by degeneration of the nervous system. It has a higher incidence in the elderly, so it is called senile dementia. Clinical manifestations are mainly memory impairment, cognitive dysfunction, mental symptoms, and behavioural abnormalities^1^. The two core pathological features of AD are amyloid plaques and neurofibrillary tangles^2^. AD features include progressive memory loss and severe cognitive decline. AD is a typical multifactorial disease, and its pathogenesis involves abnormalities in the structure and function of multiple systems^3^. Therefore, single-target drugs often do not work well. Design and develop multi-target drugs so that one drug can specifically bind to multiple targets to ensure clinical effectiveness while reducing toxicity.

Most of the cognitive symptoms of AD are caused by the depletion of cholinergic neurons in the basal forebrain, which leads to a decrease in cholinergic neurotransmission, of which acetylcholine (ACh) is the most important neurotransmitter. Various evidences indicate that Cholinesterases (ChEs) are closely related to the aetiology and symptoms of the disease. The drugs used increase cholinergic neurotransmission by inhibiting acetylcholinesterase (AChE), such as tacrine^4^, donepezil^5^, rivastigmine^6^, or galantamine^7^. The data from clinical trials of these AChE inhibitor provides convincing evidence that they have sufficient AD efficacy and reliability, as well as acceptable side effects. In recent years, the role of butyrylcholinesterase (BuChE) inhibition in AD has received increasing attention from a medicinal chemistry and clinical perspective^8^. There is evidence that BuChE may be a co-regulator of the neurotransmitter ACh activity. BuChE plays an important role in cholinergic neurotransmission and is involved in other nervous system functions and neurodegenerative diseases^9^.

One of the main causes of the course of AD is the accumulation of β-amyloid (Aβ) in the brain^10^. Aβ is a short peptide produced by the hydrolysis of amyloid precursor protein (APP) by β-secretase (BACE 1)^11^ and γ-secretase^12^. Aβ is secreted outside the cell, accumulates to form aggregates and fibrils, and finally forms amyloid deposits, which are called senile plaques^13^. Blocking the activity of BACE1 can inhibit the production of Aβ peptide. Since there are no effective therapeutic drugs in the clinic, the study of BACE1 inhibitors for therapeutic intervention in patients with Alzheimer's disease has increasingly become a research hotspot.

**Scheme 1.**
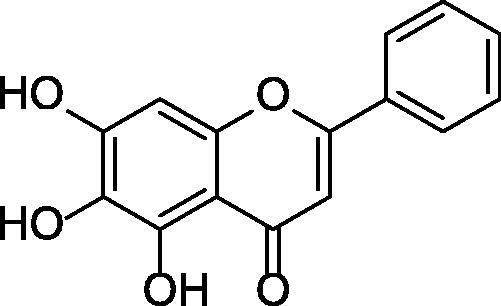
The structure of baicalein.

Studies have found that baicalein (Scheme 1) can improve neurodegenerative diseases and has the potential to treat AD^14^. However, there are three adjacent phenolic hydroxyl groups in the structure of baicalein, which are easy to form intramolecular hydrogen bonds, resulting in poor lipophilicity and hydrophilicity, and it has disadvantages such as poor solubility and low bioavailability in clinical applications. Therefore, based on the structure of baicalein, 2-arylbenzofurans were designed to explore its anti-AD activity. Benzofuran compounds have attracted much attention due to their many biological pharmacological activities, such as anti-oxidant^15^, anti-bacterial^16^, anti-fungal^17^, anti-inflammatory^18^, anti-tumour^19^, hypoglycemic^20^, anti- cholinesterase^21^, anti-monoamine oxidase^22^, and other activity. The aetiology of AD is complex, and multi-targeted drugs are superior to single-targeted drugs for this disease^23^. Recently, various benzofuran-based compounds were reported as potent acetylcholinesterase inhibitors. The natural product benzofuran skeleton can be regarded as a mimic of the indanone part of donepezil, which has a good inhibitory activity of AChE and a moderate ability to inhibit the self-mediated aggregation of Aβ_1-42_^24–25^. The benzofuran backbone has become an important pharmacophore for the design of antiviral^26^ and antibacterial agents^27^, as well as cyclin-dependent kinases (CDKs)^28^ and cholinesterase inhibitors^29^. The current study describes the preparation and *in vitro* activity of 2-arylbenzofuran derivatives as ChE inhibitors and BACE1 inhibitors. The study aims to screen ChEs/BACE1 dual target inhibitors, which have the potential to treat AD and other neurodegenerative diseases. The experiments we have performed provide an idea for the development of drug designs for therapeutic or prophylactic agents for AD.

## 2. Results and discussion

### 2.1. Molecular design

Using 40 benzaldehyde compounds and 28 methyl phenylacetate compounds as raw materials, 1120 2-arylbenzofuran compounds were designed by creating a small molecule library according to the reaction type. According to the analysis of drug-like properties, the compounds in the constructed small molecule library all meet the five rules of drug-like properties.

**Figure 1.**
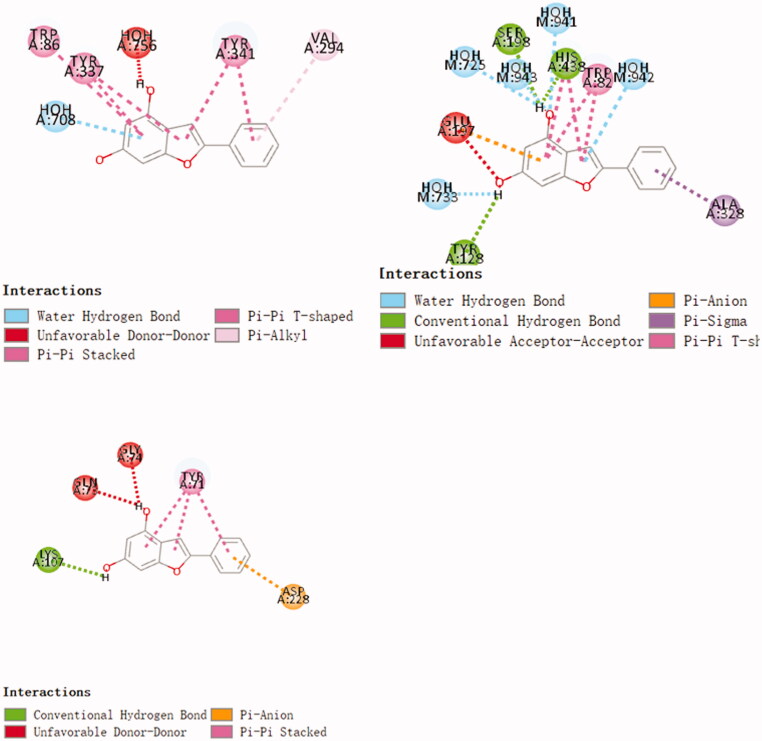
Schematic presentations of the compound 612 binding modes with AChE, BuChE and BACE1.

**Figure 2.**
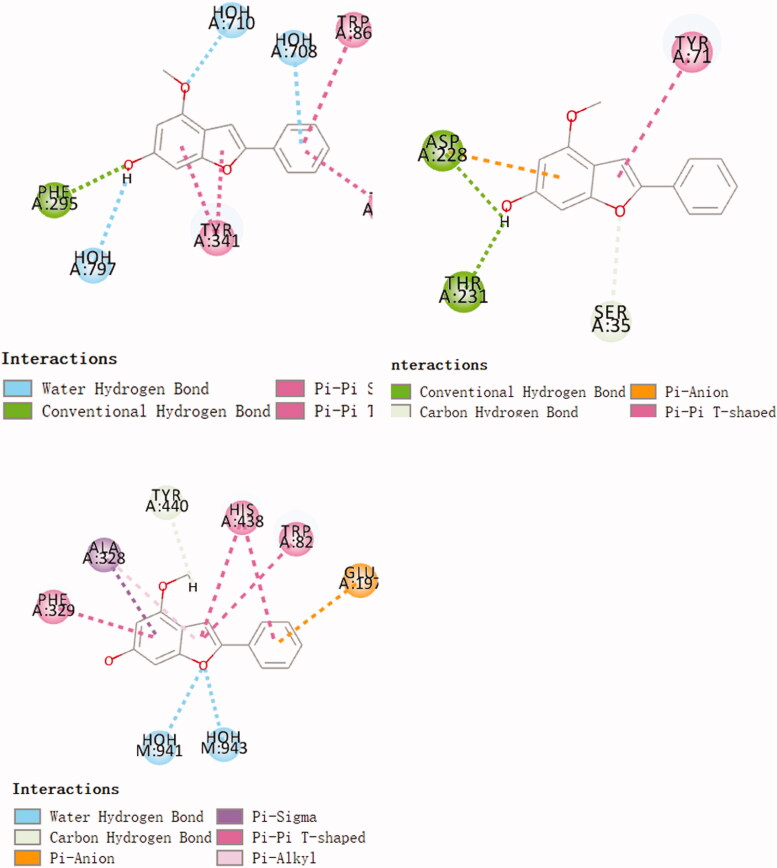
Schematic presentations of the compound 1004 binding modes with AChE, BuChE and BACE1.

**Figure 3.**
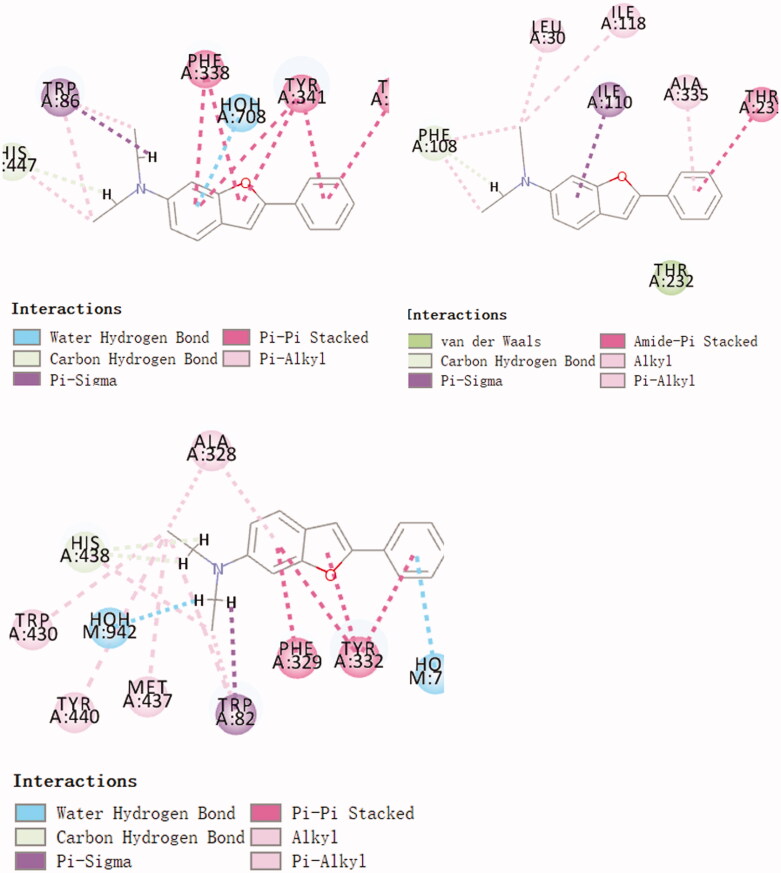
Schematic presentations of the compound 584 binding modes with AChE, BuChE and BACE1.

Use molecular docking technology to screen ChEs/BACE1 dual target inhibitors from small molecule libraries. The compounds were docked with AChE, BuChE and BACE 1, and the compound with higher scores in 3 docking times was selected as the target compound for synthesis. The final screening yielded 11 compounds with high docking scores for all three enzymes. Three randomly selected compounds and enzyme interaction patterns are displayed (Figures 1–3). Based on the comprehensive consideration of design results and synthesis cost, 22 2-arylbenzofurans have been synthesised.

### 2.2. Chemistry

**Scheme 2.**
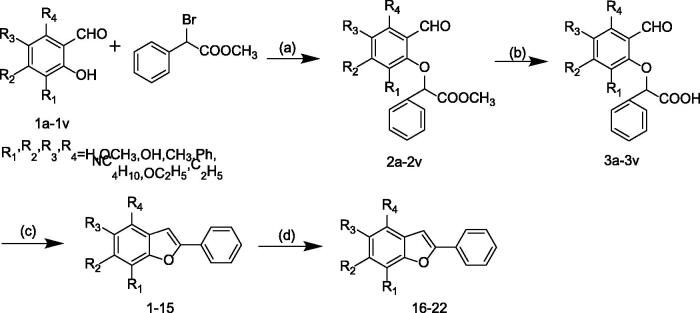
General synthetic route to 2-arylbenzofuran, reagents and conditions: (a) K_2_CO_3_, DMF, 50 °C; (b) 10%KOH, CH_3_OH, 10%HCl, 82 °C; (c) Ac_2_O, AcONa, 125 °C; (d) CH_3_CN, I_2_, Al, 90 °C.

2-Arylbenzofuran were obtained from the substituted 2-hydroxybenzaldehyde in a three-step reaction. The routes for the synthesis of 2-arylbenzofuran derivatives are shown in Scheme 2 and the final products are listed in Table 1. The synthesis method of 2-arylbenzofuran compound is based on the Droździk and co-workers’ method and slightly optimized^30^. *O*-Alkylation of the substituted 2-hydroxybenzaldehyde with methyl α-bromophenylacetate in the presence of potassium carbonate in dimethylformamide gave the corresponding methyl 2–(2-formylphenoxy)-2 -phenylacetates in high yields. Hydrolysis of the methyl 2–(2-formylphenoxy)-2 -phenylacetates to the 2–(2-formylphenoxy)-2-phenylacetic acids, followed by cyclisation of the acids to obtain the corresponding compounds 1–22. Details on the chemical and spectroscopic characterisations of compounds 1–22 were described in the Supporting Information.

**Table 1.** Compounds 1–22.

Product	R_1_	R_2_	R_3_	R_4_
1	H	H	H	H
2	OCH_3_	H	H	H
3	H	OCH_3_	H	H
4	H	H	OCH_3_	H
5	H	H	H	OCH_3_
6	H	OCH_3_	H	OCH_3_
7	CH_3_	H	H	H
8	H	CH_3_	H	H
9	H	H	CH_3_	H
10	CH_3_	H	H	CH_3_
11	H	H	Ph	Ph
12	Ph	Ph	H	H
13	H	NC_4_H_10_	H	H
14	OC_2_H_5_	H	H	H
15	H	C_2_H_5_	H	H
16	OH	H	H	H
17	H	OH	H	H
18	H	H	OH	H
19	H	H	H	OH
20	H	OH	H	OH
21	H	OH	H	OCH_3_
22	H	OCH_3_	H	OH

13, 20, 21 and 22 are new compounds.

Compared with the original method, the temperature of the reaction in the first stage is adjusted to 50 °C to prevent the reaction from proceeding excessively. Excessive reactions can affect compound yield and purity. The completion of the reaction is monitored by thin layer chromatography (TLC), not just the reaction time. Compounds with different substituents have different reaction times. Most 2-arylbenzofuran compounds can be purified by recrystallization from methanol, which is efficient and fast.

### 2.3. Biology

#### 2.3.1. *In vitro* ChE inhibitory activity

The method of Ellman et al.^31–33^ was used to evaluate the ChEs inhibitory activity of all compounds with donepezil as a standard sample. As shown in Table 2, most compounds have AChE inhibitory activity. Among all the compounds, compound 20 (IC_50_ = 0.086 ± 0.01 µmol·L^−1^) has the strongest activity, similar to the positive drug donepezil (IC_50_ = 0.079 ± 0.01 µmol·L^−1^), and far better than the natural product baicalein (IC_50_ = 0.404 ± 0.04 µmol·L^−1^). Nearly half of the compounds have BuChE inhibitory activity. In the same situation, compound 20 (IC_50_ = 16.450 ± 2.12 µmol·L^−1^) has the highest inhibitory activity, which is slightly worse than donepezil (IC_50_ = 7.100 ± 0.23 µmol·L^−1^), but still better than baicalein (IC_50_ = 31.624 ± 0.01 µmol·L^−1^). According to the results in Table 2, most of the compounds have dual ChEs inhibitory activity, and their effect may be better compared with selective ChE inhibitors. Compared with other compounds, R_4_ and R_6_ of compound 20 each have a hydroxyl substituent, which may be the reason for its high activity.

**Table 2.** Biological evaluation *in vitro.*

Product	IC_50_ value (µmol·L^−1^)		
	AChE inhibitory activity	BuChE inhibitory activity	BACE1 inhibitory activity
1	15.357 ± 7.13	>200	82.685 ± 7.49
2	80.230 ± 8.08	>200	1.499 ± 1.46
3	73.273 ± 6.92	>200	1.806 ± 0.38
4	>100	>200	>100
5	49.976 ± 2.28	41.961 ± 0.11	>100
6	7.877 ± 1.69	>200	21.452 ± 6.61
7	29.572 ± 1.12	157.202 ± 3.14	>100
8	>100	>200	0.069 ± 0.01
9	>100	>200	0.607 ± 0.29
10	19.160 ± 1.36	188.920 ± 6.25	>100
11	19.534 ± 1.73	>200	0.164 ± 0.19
12	23.501 ± 1.07	82.094 ± 1.69	0.308 ± 0.42
13	25.574 ± 1.69	>200	>100
14	25.945 ± 1.19	>200	0.445 ± 0.34
15	57.197 ± 1.93	>200	>100
16	55.294 ± 1.45	152.413 ± 4.20	11.895 ± 16.73
17	8.730 ± 1.07	143.230 ± 1.07	0.370 ± 0.29
18	31.201 ± 1.02	169.340 ± 4.13	3.634 ± 2.02
19	46.687 ± 5.24	141.267 ± 1.89	0.072 ± 0.01
20	0.086 ± 0.01	16.450 ± 2.12	0.043 ± 0.01
21	2.172 ± 0.98	53.102 ± 3.48	0.313 ± 0.12
22	3.463 ± 0.65	51.049 ± 16.15	0.392 ± 0.42
Donepezil	0.079 ± 0.01	7.100 ± 0.23	/
Baicalein	0.404 ± 0.04	31.624 ± 0.01	0.087 ± 0.03

#### 2.3.2. *In vitro* BACE1 inhibitory activity

A BACE1 activity assay kit was used to evaluate the BACE1 inhibitory activity of all compounds. As shown in Table 2, most of the compounds have good BACE1 inhibitory activity, of which 3 compounds (8, 19 and 20) show better activity than baicalein (IC_50_ = 0.087 ± 0.03 µmol·L^−1^). It can be seen that the 2-arylbenzofuran structure is a superior BACE1 inhibitor than baicalein. According to comprehensive activity research experiments, in addition to good ChEs inhibitory activity, compound 20 also has high BACE1 inhibitory activity (IC_50_ = 0.043 ± 0.01 µmol·L^−1^). We speculate from the results that the inclusion of two or more hydroxyl groups on the aromatic ring of the core of the 2-arylbenzofuran compound can increase its BACE1 inhibitory activity and also improve other activities.

#### 2.3.3. Kinetic characterisation of ChEs inhibition

**Figure 4.**
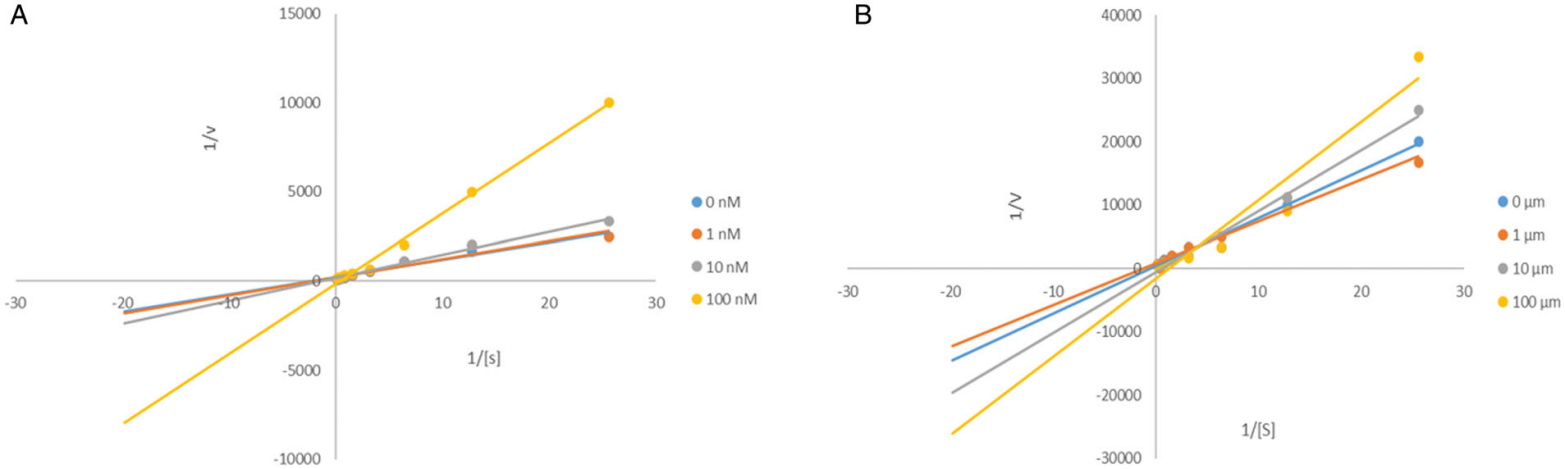
Kinetic study of the mechanism of ChEs inhibition by compound 20. Overlaid Lineweaver–Burk reciprocal plots of ChEs initial velocity at increasing substrate concentration in the absence of inhibitor and in the presence of 20 are shown. (A) A double reciprocal plot of compound 20 inhibition of AChE. (B) A double reciprocal plot of compound 20 inhibition of BuChE.

To understand the mechanism of action of these derivatives on ChEs, the compound 20, which has good inhibitory activity on ChEs, was selected for kinetic study. Figure 4 shows a graphical analysis of the steady-state suppression data for compound 20 pairs of ChEs. According to this figure, it can be judged that the inhibition mode of compound 20 on ChEs is reversible inhibition.

#### 2.3.4. Cytotoxic

**Figure 5.**
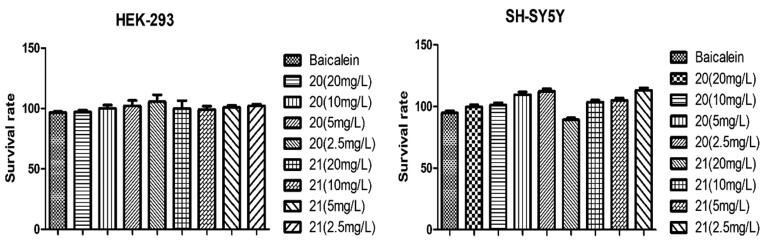
Effect of compound 20 and 21 on the survival rate of HEK-293 and SH-SY5Y cells.

In order to study the activity of the compound at the cellular level, firstly select compounds 20 and 21 with the better activity in all aspects for cytotoxicity experiment^34^. The purpose of this experiment is to study whether 2-arylbenzofurans are toxic to normal cells. The results are shown in the Figure 5, each concentration of compounds can make the survival rate of cells reach more than 90%, and it is basically non-toxic to cell growth. And there is a certain linear relationship between the cell survival rate and the concentration of the compound.

#### 2.3.5. Inhibition of reactive oxygen species in cells

**Figure 6.**
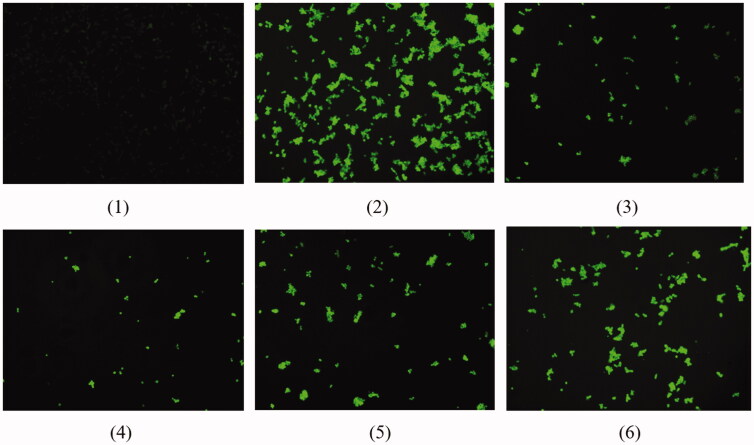
The inhibitory effect of compound 20 on ROS in cells. (1) is the ROS contained in normal cells. (2) is the sharp increase of ROS levels in the AD model cells. (3) is the inhibition of baicalein (20 mg·L^−1^) on ROS in AD model cells. (4), (5) and (6) are the inhibition of compound 20 (20, 10, 5 mg·L^−1^) on ROS in AD model cells.

The reactive oxygen species (ROS) detection kit was used to analyse the ability of compound 20 with the best activity to inhibit the level of ROS in model cells by the DCFH-DA method. As shown in Figure 6, compound 20 significantly reduces the surge of ROS levels in AD model cells, and its effect is better than that of baicalein at the same concentration. Compound 20 (5 mg·L^−1^) can significantly reduce the level of ROS in AD model cells, and as the concentration increases, the effect gets better and better. Compound 20 (20 mg·L^−1^) can reduce the level of ROS in AD model cells to be similar to normal cells. It can be seen that compound 20 can significantly improve the oxidative stress response caused by AD.

## 3. Experimental

### 3.1. Molecular design

**Scheme 3.**
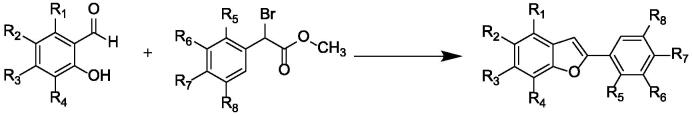
Reaction formula needed to construct the small molecule library.

Discovery Studio (DS) is a molecular simulation software in the field of life sciences, aimed at biological macromolecules and small organic molecules. A small molecule library containing a series of 2-arylbenzofuran compounds was constructed by constructing a small molecule library according to the reaction type. Draw the structure of benzaldehyde compounds and methyl phenylacetate compounds by Chemdraw. Use Chemdraw to draw the reaction formula (Scheme 3) needed to construct the small molecule library and save it in .rxn format for later use. Use Enumerate Library by Reaction in the Design and Analysis Libraries module of DS to construct a virtual combinatorial compound library by reaction type. Copy 40 benzaldehyde compounds and 28 methyl phenylacetate compounds to the same molecular window and use them as reactant options. The reaction type file selection has been saved in the .rxn format. Keep the remaining settings as default.

Compounds that meet the five rules of drug-like properties have better pharmacokinetic properties, higher bioavailability in the process of metabolism in the body, and more likely to become oral drugs. Filter the constructed small molecules through Lipinski and veber rules in Prepare or Filter Ligands built in DS. According to the analysis of drug-like properties, the compounds in the constructed small molecule library all meet the five rules of drug-like properties.

Molecular docking of the compounds into protein was performed employing Libdock rapid docking technology introduced in the DS. Three-dimensional (3 D) protein structure of AChE (PDB id: 6O4W), BuChE (PDB id: 6EP4) and BACE1 (PDB id: 4IVS) were accessed from protein data bankk. The ligand is processed first, and the prepared ligand method built into DS is used to optimise the energy of the generated ligand. Then the DS built-in prepare protein method is used for the protein required for docking. The main purpose is to hydrogenate the protein, retain the water in the protein binding pocket, and remove the original ligand.

### 3.2. Synthesis

#### 3.2.1. Materials and methods

Melting points were determined using a Thiele tube and were uncorrected. The ^1^H NMR and ^13 ^C NMR spectra were recorded with a Bruker AM-600 spectrometer (Billercia, MA, USA) with TMS as the internal standard. Chemical shifts were reported at room temperature on a scale (ppm) with CDCl_3_ or DMSO-d_6_ as the solvents and J values are given in Hertz. Mass spectra were obtained with an Agilent Trap VL LC/MS spectrometer (Santa Clara, CA, USA). The absorbance was recorded by RZ-9618 Microplate Reader. Unless otherwise noted, all solvents and reagents were commercially available and used without further purification.

#### 3.2.2. General method for synthesis of compounds 2a–2v

Taking the synthesis of methyl 2–(2-formyl-6-methoxyphenoxy)-2-phenylacetate as an example. Other phenylacetates were obtained using the same procedures. A mixture of methyl 2-bromo-2-phenylacetate (1.08 g, 4.7 mmol), *o*-Vanillin (0.72 g, 4.7 mmol), K_2_CO_3_ (0.76 g, 5.5 mmol) and DMF (10 ml) was stirred and heated at 92–94 °C. After the TLC detection reaction was completed, the mixture was cooled, poured onto ice/H_2_O (150 ml) and the mixture was kept at 4 °C overnight. After that, the precipitate was filtered, washed with water and dried, and recrystallized from methanol to obtain white crystals (yield: 85.10%).

#### 3.2.3. General method for synthesis of compounds 3a–3v

Taking the synthesis of 2–(2-formyl-6-methoxyphenoxy)-2-phenylacetic acid as an example. Other phenylacetic acids were obtained using the same procedures. A mixture of methyl 2–(2-formyl-6-methoxyphenoxy)-2-phenylacetate (2.10 g, 7 mmol), 10% aq KOH (30 ml) and methanol (2 ml) was stirred and heated at 80–82 °C for 2 h. After the mixture was cooled to room temperature, it was poured onto ice/H_2_O (150 ml), and 10% aqueous HCl was added with stirring until precipitation appeared. The precipitate was filtered off, washed several times with H_2_O and dried. The crude product was obtained as a solid (yield: 78.57%).

#### 3.2.4. General method for synthesis of compounds 1–15

Taking the synthesis of 7-methoxy-2-phenylbenzofuran as an example. Compounds 2–15 were obtained using the same procedures. A mixture of 2–(2-formyl-6-methoxyphenoxy)-2-phenylacetic acid (0.94 g, 3.3 mmol), anhydrous AcONa (2.71 g, 33 mmol) and Ac_2_O (35 ml) was stirred and heated at 120–125 °C for 4 h. Then, the mixture was cooled and poured onto ice/H_2_O (200 ml) and left at refrigerator for 12 h. The precipitate was filtered off, washed several times with cooled H_2_O and dried. The crude product was recrystallized from *n*-hexane (yield: 90.91%).

#### 3.2.5. General method for synthesis of compounds 16–22

Taking the synthesis of 2-phenylbenzofuran-7-ol as an example. Compounds 17–22 were obtained using the same procedures. Add 150 ml of acetonitrile solution to the three-necked reaction flask, add iodine (1.27 g, 5 mmol) and aluminium powder (0.50 g, 18.5 mmol), reflux for 3 h, cool to room temperature, add 0.56 g (2.5 mmoL) of compound 1, and heat to reflux for reaction, TLC monitors the progress of the reaction, and the reaction is complete after 24 h. The reaction solution was concentrated, and the concentrated solution was washed with 5% sodium bisulphite to remove excess iodine. After acidification with hydrochloric acid, the aqueous solution was extracted with ethyl acetate. The organic layer was concentrated and evaporated to dryness to obtain a yellow product (yield: 72%).

##### 3.2.5.1. 2-Phenylbenzofuran (1)

White solid, yield 65.14%. ^1^H NMR (600 MHz, CDCl_3_) *δ* 7.90 (m, 2H, Ar), 7.61 (d, *J* = 7.6 Hz, 1H, Ar), 7.55 (d, *J* = 8.1 Hz, 1H, Ar), 7.47 (t, 2H, Ar), 7.38 (t, 1H, Ar), 7.31 (dd, 1H, Ar), 7.25 (d, *J* = 7.3 Hz, 1H, Ar), 7.05 (s, 1H, Ar). ^13 ^C NMR (150 MHz, CDCl_3_) *δ* 155.90 (s), 154.87 (s), 130.47 (s), 129.20 (s), 128.77 (s), 128.53 (s), 124.92 (s), 124.24 (s), 122.91 (s), 120.88 (s), 111.16 (s), 101.28 (s). MS: *m/z* (%) [M + Na]^+^ 217.6.

##### 3.2.5.2. 7-Methoxy-2-phenylbenzofuran (2)

White solid, yield 90.91%. ^1^H NMR (600 MHz, CDCl_3_) *δ* 7.92 (m, 2H, Ar), 7.46 (t, 2H, Ar), 7.36 (t, 1H, Ar), 7.19 (m, 2H, Ar), 7.03 (s, 1H, Ar), 6.83 (d, *J* = 7.7 Hz, 1H, Ar), 4.07 (s, 3H, OCH_3_). ^13 ^C NMR (150 MHz, CDCl_3_) *δ* 156.05 (s), 145.31 (s), 144.11 (s), 130.91 (s), 130.31 (s), 128.69 (s), 128.53 (s), 125.02 (s), 123.57 (s), 113.31 (s), 106.69 (s), 101.61 (s), 56.13 (s). MS: *m/z* (%) [M + Na]^+^ 247.5, [2M + Na]^+^ 471.5.

##### 3.2.5.3. 6-Methoxy-2-phenylbenzofuran (3)

White solid, yield 79.4%. ^1^H NMR (600 MHz, CDCl_3_) *δ* 7.83 (m, 2H, Ar), 7.45 (m, 3H, Ar), 7.34 (t, 1H, Ar), 7.09 (d, *J* = 1.8 Hz, 1H, Ar), 6.97 (s, 1H, Ar), 6.89 (dd, *J* = 8.5, 2.2 Hz, 1H, Ar), 3.89 (s, 3H, OCH_3_). ^13 ^C NMR (150 MHz, CDCl_3_) *δ* 158.05 (s), 155.89 (s), 155.12 (s), 130.69 (s), 128.73 (s), 128.03 (s), 124.43 (s), 122.53 (s), 120.97 (s), 111.95 (s), 101.13 (s), 95.85 (s), 55.72 (s). MS: *m/z* (%) [M + Na]^+^ 247.2, [2M + Na]^+^ 471.2.

##### 3.2.5.4. 5-Methoxy-2-phenylbenzofuran (4)

White solid, yield 83.3%. ^1^H NMR (600 MHz, CDCl_3_) *δ* 7.86 (m, 2H, Ar), 7.44 (dd, 3H, Ar), 7.36 (t, 1H, Ar), 7.06 (d, *J* = 2.6 Hz, 1H, Ar), 6.98 (s, 1H, Ar), 6.91 (dd, *J* = 8.9, 2.6 Hz, 1H, Ar), 3.88 (s, 3H, OCH_3_). ^13 ^C NMR (150 MHz, CDCl_3_) *δ* 156.68 (s), 156.05 (s), 149.92 (s), 130.53 (s), 129.75 (s), 128.75 (s), 128.48 (s), 124.83 (s), 112.96 (s), 111.58 (s), 103.29 (s), 101.45 (s), 55.88 (s). MS: *m/z* (%) [M + Na]^+^ 247.6, [2M + Na]^+^ 471.6.

##### 3.2.5.5. 4-Methoxy-2-phenylbenzofuran (5)

White solid, yield 71.6%. ^1^H NMR (600 MHz, CDCl_3_) *δ* 7.87 (dd, *J* = 8.3, 1.2 Hz, 2H, Ar), 7.46 (t, 2H, Ar), 7.35 (t, 1H, Ar), 7.23 (t, 1H, Ar), 7.19 (d, *J* = 8.3 Hz, 1H, Ar), 7.15 (d, *J* = 0.7 Hz, 1H, Ar), 6.69 (d, *J* = 7.8 Hz, 1H, Ar), 3.98 (s, 3H, OCH_3_). ^13 ^C NMR (150 MHz, CDCl_3_) *δ* 156.04 (s), 154.61 (s), 153.41 (s), 130.53 (s), 128.74 (s), 128.26 (s), 124.94 (s), 124.71 (s), 119.50 (s), 104.45 (s), 103.28 (s), 98.76 (s), 55.58 (s). MS: *m/z* (%) [M + Na]^+^ 247.7, [2M + Na]^+^ 471.7.

##### 3.2.5.6. 4,6-Dimethoxy-2-phenylbenzofuran (6)

White solid, yield 94.1%. ^1^H NMR (600 MHz, CDCl_3_) *δ* 7.81 (dd, *J* = 8.3, 1.1 Hz, 2H, Ar), 7.43 (m, 2H, Ar), 7.31 (m, 1H, Ar), 7.05 (d, *J* = 0.7 Hz, 1H, Ar), 6.72 (m, 1H, Ar), 6.35 (d, *J* = 1.9 Hz, 1H, Ar), 3.94 (s, 3H, OCH_3_), 3.88 (s, 3H, OCH_3_). ^13 ^C NMR (150 MHz, CDCl_3_) *δ* 159.16 (s), 156.54 (s), 153.65 (s), 153.45 (s), 130.75 (s), 128.70 (s), 127.72 (s), 124.19 (s), 113.22 (s), 98.72 (s), 94.29 (s), 88.20 (s), 55.76 (s), 55.55 (s). MS: *m/z* (%) [M + H]^+^ 255.6, [M + Na]^+^ 277.7, [2M + Na]^+^ 531.7.

##### 3.2.5.7. 7-Methyl-2-phenylbenzofuran (7)

White solid, yield 79.2%. ^1^H NMR (600 MHz, CDCl_3_) *δ* 7.91 (dd, *J* = 8.3, 1.2 Hz, 2H, Ar), 7.46 (m, 3H, Ar), 7.37 (m, 1H, Ar), 7.16 (t, 1H, Ar), 7.11 (d, *J* = 7.2 Hz, 1H, Ar), 7.04 (s, 1H, Ar), 2.62 (s, 3H, CH_3_). ^13 ^C NMR (150 MHz, CDCl_3_) *δ* 155.51 (s), 153.89 (s), 130.67 (s), 128.69 (d, *J* = 11.6 Hz), 128.40 (s), 125.19 (s), 124.87 (s), 122.94 (s), 121.40 (s), 118.33 (s), 101.56 (s), 15.04 (s). MS: *m/z* (%) [M + H]^+^ 209.6, [M + Na]^+^ 231.1.

##### 3.2.5.8. 6-Methyl-2-phenylbenzofuran (8)

White solid, yield 85.32%. ^1^H NMR (600 MHz, CDCl_3_) *δ* 7.87 (dd, *J* = 8.4, 1.2 Hz, 2H, Ar), 7.46 (m, 3H, Ar), 7.36 (m, 2H, Ar), 7.08 (dd, *J* = 7.9, 0.7 Hz, 1H, Ar), 7.00 (d, *J* = 0.8 Hz, 1H, Ar), 2.51 (s, 3H, CH_3_). ^13 ^C NMR (150 MHz, CDCl_3_) *δ* 155.33 (s), 155.31 (s), 134.54 (s), 130.67 (s), 128.73 (s), 128.25 (s), 126.67 (s), 124.73 (s), 124.33 (s), 120.34 (s), 111.41 (s), 101.16 (s), 21.74 (s). MS: *m/z* (%) [M + Na]^+^ 231.6.

##### 3.2.5.9. 5-Methyl-2-phenylbenzofuran (9)

White solid, yield 91.11%. ^1^H NMR (600 MHz, CDCl_3_) *δ* 7.88 (m, 2H, Ar), 7.44 (dd, 3H, Ar), 7.37 (dd, 2H, Ar), 7.12 (dd, *J* = 8.3, 1.3 Hz, 1H, Ar), 6.97 (s, 1H, Ar), 2.47 (s, 3H, CH_3_). ^13 ^C NMR (150 MHz, CDCl_3_) *δ* 155.97 (s), 153.31 (s), 132.31 (s), 130.61 (s), 129.29 (s), 128.73 (s), 128.39 (s), 125.51 (s), 124.83 (s), 120.71 (s), 110.63 (s), 101.06 (s), 21.33 (s). MS: *m/z* (%) [M + H]^+^ 209.1, [M + Na]^+^ 231.1.

##### 3.2.5.10. 4,7-Dimethyl-2-phenylbenzofuran (10)

White solid, yield 58.0%. ^1^H NMR (600 MHz, CDCl_3_) *δ* 7.92 (dd, *J* = 8.3, 1.1 Hz, 2H, Ar), 7.48 (t, 2H, Ar), 7.37 (m, 1H, Ar), 7.06 (s, 1H, Ar), 7.01 (d, *J* = 7.4 Hz, 1H, Ar), 6.96 (d, *J* = 7.4 Hz, 1H, Ar), 2.58 (s, 3H, CH_3_), 2.54 (s, 3H, CH_3_). ^13 ^C NMR (150 MHz, CDCl_3_) *δ* 155.02 (s), 153.60 (s), 130.80 (s), 128.71 (s), 128.43 (s), 128.25 (s), 128.08 (s), 125.07 (s), 124.79 (s), 123.10 (s), 118.58 (s), 100.29 (s), 18.34 (s), 14.81 (s). MS: *m/z* (%) [M + H]^+^ 223.2, [M + Na]^+^ 245.3, [2M + Na]^+^ 467.3.

##### 3.2.5.11. 2-Phenylnaphtho[2,1-b]furan (11)

White solid, yield 81.8%. ^1^H NMR (600 MHz, CDCl_3_) *δ* 8.08 (d, *J* = 8.2 Hz, 1H, Ar), 7.85 (m, 3H, Ar), 7.62 (m, 2H, Ar), 7.51 (m, 1H, Ar), 7.41 (m, 4H, Ar), 7.16 (s, 1H, Ar). ^13 ^C NMR (150 MHz, CDCl_3_) *δ* 155.37 (s), 152.35 (s), 130.63 (s), 130.41 (s), 128.83–128.79 (d, *J* = 6.1 Hz), 128.25 (s), 127.59(s), 126.25 (s), 125.16 (s), 124.64 (s), 124.55–124.52 (d *J* = 4.5 Hz), 123.44 (s), 112.27 (s), 100.43 (s). MS: *m/z* (%) [M + H]^+^ 245.6, [M + Na]^+^ 267.5.

##### 3.2.5.12. 2-Phenylnaphtho[1,2-b]furan (12)

White solid, yield 83.2%. ^1^H NMR (600 MHz, CDCl_3_) *δ* 8.43 (d, *J* = 8.2 Hz, 1H, Ar), 7.97 (m, 3H, Ar), 7.64 (m, 3H, Ar), 7.51 (m, 3H, Ar), 7.38 (m, 1H, Ar), 7.17 (s, 1H, Ar). ^13 ^C NMR (150 MHz, CDCl_3_) *δ* 155.30 (s), 150.29 (s), 131.47 (s), 130.72 (s), 128.81 (s), 128.41 (s), 128.21 (s), 126.33 (s), 125.02 (s), 124.80 (s), 124.65 (s), 123.61 (s), 121.33 (s), 120.01 (s), 119.54 (s), 102.45 (s). MS: *m/z* (%) [M + H]^+^ 245.3, [M + Na]^+^ 267.1.

##### 3.2.5.13. N, N-diethyl-2-phenylbenzofuran-6-amine (13)

White solid, yield 90.1%. ^1^H NMR (600 MHz, DMSO-d_6_) *δ* 7.80 (dd, *J* = 8.3, 1.1 Hz, 2H, Ar), 7.44 (t, 2H, Ar), 7.39 (d, *J* = 8.6 Hz, 1H, Ar), 7.30 (t, 1H, Ar), 7.21 (d, *J* = 0.6 Hz, 1H, Ar), 6.82 (d, *J* = 1.8 Hz, 1H, Ar), 6.69 (dd, *J* = 8.7, 2.2 Hz, 1H, Ar), 3.38 (q, *J* = 7.0 Hz, 4H, CH_2_), 1.11 (t, 6H, CH_3_). ^13 ^C NMR (150 MHz, DMSO-d_6_) *δ* 156.73 (s), 152.18 (s), 146.29 (s), 130.48 (s), 128.90 (s), 127.54 (s), 123.63 (s), 121.24 (s), 117.69 (s), 109.64 (s), 101.96 (s), 93.46 (s), 44.19 (s), 12.39 (s). HR-MS: *m/z* (%) [M + H]^+^ 266.1215.

##### 3.2.5.14. 7-Ethoxy-2-phenylbenzofuran (14)

White solid, yield 79.1%. ^1^H NMR (600 MHz, CDCl_3_) *δ* 7.92 (dd, *J* = 8.3, 1.1 Hz, 2H, Ar), 7.46 (t, 2H, Ar), 7.37 (m, 1H, Ar), 7.17 (m, 2H, Ar), 7.03 (s, 1H, Ar), 6.83 (dd, *J* = 7.8, 0.9 Hz, 1H, Ar), 4.35 (q, *J* = 7.0 Hz, 2H, CH_2_), 1.56 (t, 3H, CH_3_). ^13 ^C NMR (150 MHz, CDCl_3_) *δ* 155.93 (s), 144.55 (s), 144.37 (s), 131.02 (s), 130.37 (s), 128.68 (s), 128.48 (s), 125.03 (s), 123.55 (s), 113.23 (s), 108.18 (s), 101.62 (s), 64,74 (s), 15.00 (s). MS: *m/z* (%) [M + H]^+^ 239.3, [M + Na]^+^ 261.1.

##### 3.2.5.15. 6-Ethyl-2-phenylbenzofuran (15)

Yellow solid, yield 78.8%. ^1^H NMR (600 MHz, CDCl_3_) *δ* 7.86 (dd, *J* = 8.2, 1.0 Hz, 2H, Ar), 7.49 (d, *J* = 7.9 Hz, 1H, Ar), 7.46 (dd, 2H, Ar), 7.36 (m, 2H, Ar), 7.10 (dd, *J* = 7.9, 1.1 Hz, 1H, Ar), 7.00 (d, *J* = 0.6 Hz, 1H, Ar), 2.79 (t, *J* = 7.6 Hz, 2H, CH_2_), 1.31 (t, 3H, CH_3_). ^13 ^C NMR (150 MHz, CDCl_3_) *δ* 155.40 (d, *J* = 12.2 Hz), 141.22 (s), 130.69 (s), 128.73 (s), 128.26 (s), 126.89 (s), 124.73 (s), 123.30 (s), 120.44 (s), 110.15 (s), 101.17 (s), 29.14 (s), 15.99 (s). MS: *m/z* (%) [M + H]^+^ 223.5, [M + Na]^+^ 245.4.

##### 3.2.5.16. 2-Phenylbenzofuran-7-ol (16)

Light yellow solid, yield 72%. ^1^H NMR (600 MHz, CDCl_3_) *δ* 7.87 (m, 2H, Ar), 7.46 (t, 2H, Ar), 7.38 (t, 1H, Ar), 7.17 (dd, *J* = 7.7, 0.9 Hz, 1H, Ar), 7.12 (t, 1H, Ar), 7.04 (s, 1H, Ar), 6.86 (dd, *J* = 7.7, 0.8 Hz, 1H, Ar). ^13 ^C NMR (150 MHz, CDCl_3_) *δ* 156.04 (s), 143.10 (s), 140.77 (s), 130.79 (s), 130.21 (s), 128.74 (d, *J* = 17.8 Hz), 124.93 (s), 123.91 (s), 113.22 (s), 110.71 (s), 102.05 (s). MS: *m/z* (%) [M + Na]^+^ 233.2, [2M + Na]^+^ 443.2.

##### 3.2.5.17. 2-Phenylbenzofuran-6-ol (17)

Light yellow solid, yield 76.6%. ^1^H NMR (600 MHz, CDCl_3_) *δ* 7.82 (dd, *J* = 8.3, 1.1 Hz, 2H, Ar), 7.43 (m, 3H, Ar), 7.33 (m, 1H, Ar), 7.03 (d, *J* = 1.9 Hz, 1H, Ar), 6.96 (d, *J* = 0.6 Hz, 1H, Ar), 6.80 (dd, *J* = 8.3, 2.2 Hz, 1H, Ar). ^13 ^C NMR (150 MHz, CDCl_3_) *δ* 155.75 (s), 155.28 (s), 153.59 (s), 130.59 (s), 128.73 (s), 128.10 (s), 124.48 (s), 122.87 (s), 121.12 (s), 112.03 (s), 101.11 (s), 98.28 (s). MS: *m/z* (%) [M + Na]^+^ 233.3, [2M + Na]^+^ 443.3.

##### 3.2.5.18. 2-Phenylbenzofuran-5-ol (18)

Light yellow solid, yield 84.3%. ^1^H NMR (600 MHz, DMSO-d_6_) *δ* 9.22 (s, 1H, OH), 7.87 (dd, *J* = 8.3, 1.2 Hz, 2H, Ar), 7.49 (t, 2H, Ar), 7.39 (ddd, 2H, Ar), 7.28 (d, *J* = 0.8 Hz, 1H, Ar), 6.95 (d, *J* = 2.4 Hz, 1H, Ar), 6.76 (dd, *J* = 8.8, 2.5 Hz, 1H, Ar). ^13 ^C NMR (150 MHz, DMSO-d_6_) *δ* 155.54 (s), 153.55 (s), 148.48 (s), 129.98 (s), 129.58 (s), 129.01 (s), 128.64 (s), 124.47 (s), 113.35 (s), 111.32 (s), 105.37 (s), 101.98 (s). MS: *m/z* (%) [M + Na]^+^ 233.7, [2M + Na]^+^ 443.7.

##### 3.2.5.19. 2-Phenylbenzofuran-4-ol (19)

Light yellow solid, yield 83.2%. ^1^H NMR (600 MHz, DMSO-d_6_) *δ* 10.03 (s, 1H, OH), 7.88 (dd, *J* = 8.3, 1.1 Hz, 2H, Ar), 7.48 (m, 2H, Ar), 7.41 (d, *J* = 0.8 Hz, 1H, Ar), 7.38 (m, 1H, Ar), 7.11 (t, 1H, Ar), 7.06 (d, *J* = 8.2 Hz, 1H, Ar), 6.64 (dd, *J* = 7.7, 0.7 Hz, 1H, Ar). ^13 ^C NMR (150 MHz, DMSO-d_6_) *δ* 155.84 (s), 153.21 (s), 151.31 (s), 129.94 (s), 129.01 (s), 128.43 (s), 125.50 (s), 124.36 (s), 118.19 (s), 108.02 (s), 102.29 (s), 99.63 (s). MS: *m/z* (%) [M + Na]^+^ 233.1.

##### 3.2.5.20. 2-Phenylbenzofuran-4,6-diol (20)

Lilac solid, yield 62.8%. ^1^H NMR (600 MHz, CDCl_3_) *δ* 7.78 (d, *J* = 7.6 Hz, 2H, Ar), 7.42 (t, 3H, Ar), 6.99 (s, 1H, Ar), 6.63 (s, 1H, Ar), 6.25 (d, *J* = 1.6 Hz, 1H, Ar). ^13 ^C NMR (150 MHz, CDCl_3_) *δ* 156.88 (s), 154.38 (s), 154.00 (s), 149.28 (s), 130.49 (s), 128.73 (s), 127.98 (s), 124.33 (s), 112.33 (s), 98.03 (s), 97.88 (s), 91.52 (s). HR-MS: *m/z* (%) [M + H]^+^ 227.1260.

##### 3.2.5.21. 4-Methoxy-2-phenylbenzofuran-6-ol (21)

Purple solid, yield 71.1%. ^1^H NMR (600 MHz, CDCl_3_) *δ* 7.79 (dd, *J* = 8.3, 1.0 Hz, 2H, Ar), 7.42 (t, 2H, Ar), 7.31 (dd, 1H, Ar), 7.04 (d, *J* = 0.5 Hz, 1H, Ar), 6.65 (d, *J* = 0.9 Hz, 1H, Ar), 6.28 (d, *J* = 1.8 Hz, 1H, Ar), 3.92 (s, 3H, OCH_3_). ^13 ^C NMR (150 MHz, CDCl_3_) *δ* 156.35 (s), 154.59 (s), 153.67 (d, *J* = 18.9 Hz), 130.65 (s), 128.70 (s), 127.80 (s), 124.24 (s), 113.25 (s), 98.69 (s), 94.17 (s), 91.20 (s), 55.62 (s). HR-MS: *m/z* (%) [M + H]^+^ 241.0574.

##### 3.2.5.22. 6-Methoxy-2-phenylbenzofuran-4-ol (22)

Lilac solid, yield 50.3%. ^1^H NMR (600 MHz, CDCl_3_) *δ* 7.79 (m, 2H, Ar), 7.42 (d, *J* = 8.0 Hz, 2H, Ar), 7.32 (dd, *J* = 10.1, 3.8 Hz, 1H, Ar), 7.02 (d, *J* = 0.5 Hz, 1H, Ar), 6.72 (d, *J* = 1.1 Hz, 1H, Ar), 6.33 (d, *J* = 1.9 Hz, 1H, Ar), 3.85 (s, 3H, OCH_3_). ^13 ^C NMR (150 MHz, CDCl_3_) *δ* 158.79 (s), 157.02 (s), 153.95 (s), 149.15 (s), 130.55 (s), 128.72 (s), 127.92 (s), 124.28 (s), 112.35 (s), 97.95 (s), 97.76 (s), 89.02 (s), 55.79 (s). HR-MS: *m/z* (%) [M + H]^+^ 241.0575.

### 3.3. Biological activity

#### 3.3.1. *In vitro* ChEs inhibitory activity

The ChEs inhibitory activity of the synthesised 2-arylbenzofuran compounds was evaluated by Ellman's method^31^ with slight modifications. Acetylthiocholine iodide and S-butyrylthiocholine iodide were used as substrates. 5,5′-Dithio bis-(2-nitrobenzoic acid) (DTNB) was used as a reagent. Donepezil was used as a positive control. Add 120 µL phosphate buffer solution (0.1 mol·L^−1^, pH = 8.0, PBS), 20 µL DTNB (3.3 mmol·L^−1^ in 0.1 mol·L^−1^ PBS, pH = 8.0), 20 µL AChE solution (0.2 U·mL^−1^ in 0.1 mol·L^−1^ PBS, pH = 8.0), and 20 µL sample solutions of different concentrations to the 96-well plate in sequence, shake well, and then incubate at 37 °C for 5 min. Then, add 20 µL of substrate (5 mmol·L^−1^ in 0.1 mol·L^−1^ PBS, pH = 8.0), shake well, and incubate at 37 °C for 20 min. Set the sample solution to 4 concentration gradients, and repeat the experiment 3 times. Use a microplate reader to measure the absorbance of the sample at 412 nm, and calculate the ChEs inhibition rate and IC_50_ value of each sample according to the formula.

$$\text{ChEs}\;\text{inhibitory}\;\text{effect}\;\left(\%\right)\;=\;\left[A_{0}-\;\left(A_{1}-{\;A}_{2}\right)\right]/A_{0}\times 100\%,$$





where A_0_ is the absorbance of the blank group; A_1_ is the absorbance of the sample group; A_2_ is the absorbance of the sample blank group.

#### 3.3.2. *In vitro* BACE1 inhibitory activity

Set the fluorometer on well plate reader mode with excitation at 320 nm and emission at 405 nm. Bring all components (except the BACE1 Enzyme Solution) to room temperature. Add components to a fluorometer 96 well plate according to Table 3. Mix well by gentle pipetting. Add the BACE1 Enzyme Solution just before reading. Read the fluorescence immediately after adding the enzyme. This is “time zero” reading. The signal in the wells could increase between the addition of enzyme and this initial reading. Cover the plate with parafilm and incubate at 37 °C for 2 h. Read the signal at “time zero” plus 2 h. The plate should be at room temperature before reading. After the readings are made, add 40 µL of Stop Solution. The addition of the Stop Solution will stabilise the signal for at least 24 h. The final reading of each well is the “2 h” reading minus “time zero” reading.

**Table 3.** Reaction scheme.

Assay description	Negative control	Positive control	Inhibition
Fluorescent assay buffer	80 µL	78 µL	78-X µL
BACE1 substrate solution, 50 µM	20 µL	20 µL	20 µL
BACE1 enzyme solution, 0.3 unit/µL	–	2 µL	2 µL
Inhibitor solution	–	–	X µL
Total	100 µL	100 µL	100 µL

The Positive Control group represents 100% enzyme activity. Calculate the BACE1 inhibition rate and IC_50_ value of each sample according to the formula.

$$\text{BACE1 inhibitory effect }\left(\%\right)\;=\;\left[\text{1 }–{\text{ A}}_{\text{2}}/{\text{A}}_{\text{1}}\right]\;\times \text{ 1}00\%,$$

where A_1_ is the reading of the Positive Control group; A_2_ is the reading of the Inhibition group.

#### 3.3.3. Kinetic characterisation of ChEs inhibition

The compound that showed better inhibitory activity against ChEs was selected for kinetic measurements. To obtain the mechanism of action of compound, reciprocal plots of velocity versus substrate were constructed at different substrate solution concentrations by using Ellman's method^31^. The experiment were measured at eight different substrate solution concentrations (0.039, 0.078, 0.156, 0.312, 0.625, 1.25, 2.5, 5 mM) and four different concentrations of target compound (0, 1, 10 and 100 nM). To a 96-well plate, phosphate buffer solution, 5,5-dithiobis-2-nitrobenzoic acid, AChE solution and target compound were added sequentially, shaken well, and incubated at 37 °C for 5 min. Then add the substrate, shake well, immediately use the microplate reader to measure the absorbance of the sample at 412 nm, and repeat the experiment three times. Lineweaver–Burk plot was made based on the reaction rate and substrate concentration to determine the type of inhibition of the ChEs by the compound.

#### 3.3.4. Cytotoxicity test

The cytotoxicity test was carried out using the MTT assay^34^. The detection principle is that the succinate dehydrogenase in the mitochondria of living cells can reduce the exogenous MTT to water-insoluble blue-purple crystal formazan and deposit it in the cells, while dead cells have no such function. Dimethyl sulfoxide (DMSO) can dissolve formazan in cells, and its absorbance value (OD value) is measured at 490 nm wavelength with a microplate reader, which can indirectly reflect the number of living cells. The greater the OD value, the stronger the cell viability and the lower the toxicity of the compound.

Collect adherent cells, adjust the concentration of cell suspension, add 100 µL to each well, and pave the plate so that the density of the cells to be tested is 5000–10,000 per well. Pave the plate in the afternoon of the previous day, add compounds in the morning of the next day, set up 5 gradients, each hole is 100 µL, and set up 3 duplicate holes. Incubate in an incubator for 48 h, add 10 µL MTT solution to each well, and continue to incubate for 4 h. After terminating the culture, aspirate the culture medium in the wells, add 150ul DMSO to each well, and place on a shaker to shake at low speed for 10 min to fully dissolve the crystals. Measure the OD value of each well at 490 nm of the microplate reader, and calculate the cell survival rate according to the formula.

$$\text{Cell survival rate }\left(\%\right)\;={\text{ A}}_{\text{1}}/{\text{A}}_{\text{2}}\times \text{ 1}00\%,$$

where A_1_ is the OD value of the sample group; A_2_ is the OD value of the blank group.

#### 3.3.5. Inhibition of reactive oxygen species in cells

Reactive oxygen species (ROS) include superoxide free radicals, hydrogen peroxide, and its downstream products peroxides and hydroxylates, etc., and participate in cell growth and proliferation, development and differentiation, ageing and apoptosis, as well as many physiological and pathological processes^35^. The ROS detection kit uses the fluorescent probe DCFH-DA for reactive oxygen detection. Dilute DCFH-DA with DMEM medium at a ratio of 1:1500. Remove the cell culture medium, add DCFH-DA diluted in an appropriate volume, and incubate for 20 min in a cell incubator. The cells were washed three times with DMEM medium to sufficiently remove DCFH-DA that did not enter the cells. The cells of each group were directly observed with a laser confocal microscope.

## 4. Conclusion

In summary, a series of 2-arylbenzofuran derivatives have been designed, synthesised and evaluated as multi-target anti-AD drugs, which have ChE inhibitory activity and BACE1 inhibitory activity. According to the test results of inhibition of ChEs, most of the compounds have dual ChEs inhibitory activity, and their effect may be better compared with selective ChE inhibitors. Compound 20 has superior BACE1 inhibitory activity than other compounds. Compared with other compounds, R_4_ and R_6_ of compound 20 each have a hydroxyl substituent, which may be the reason for its high activity. We selected compound 20 to study the kinetics of ChEs inhibition. According to kinetic experiments, the type of ChEs inhibitory effect of compound 20 is reversible, indicating that our design strategy is reasonable. The compound has extremely low toxicity to normal cells and has almost no effect. Compound 20, which has the best activity in all aspects, can significantly reduce the level of ROS in AD model cells. Our research shows that the number and position of hydroxyl groups on the aromatic ring are important. Among the tested compounds, the two hydroxyl substituents in R_4_ and R_6_ have the strongest inhibitory effect on ChEs. We also speculate that the meta-substitution of the hydroxymethoxy group promotes the increase of the activity of the compound. In conclusion, our experiments on 2-arylbenzofuran provide an idea for the development of drug design for treatment or prophylaxis for AD.

## Supplementary Material

Supplemental Material
