# Supplementary material for: Synthesis and biological evaluation of 2-arylbenzofuran derivatives as potential anti-Alzheimer’s disease agents
Source: J Enzyme Inhib Med Chem. 2021 Jun 16;36(1):1345–55. doi: 10.1080/14756366.2021.1940993 (PMC8765280; doi:10.1080/14756366.2021.1940993)

Synthesis and biological evaluation of 2-arylbenzofuran derivatives as potential anti-Alzheimer's disease agents

Yinling Yun[a], Yuhang Miao[a], Xiaoya Sun[a], Qiang Zhang[a], Jie Sun[a*] and Xiaojing Wang[a*]

a. Institute of Materia Medica，Shandong First Medical University & Shandong Academy of Medical Sciences，Jinan 250117，Shandong，China

***** Correspondence: sunjie310@126.com(J.S.); xiaojing6@gmail.com (X.J.W.).

^{1}H NMR (600 MHz, $CDCl_3$) and ^{13}C NMR (150 MHz, $CDCl_3$) of compounds **1-22**.

compound 1 ^{1}H NMR

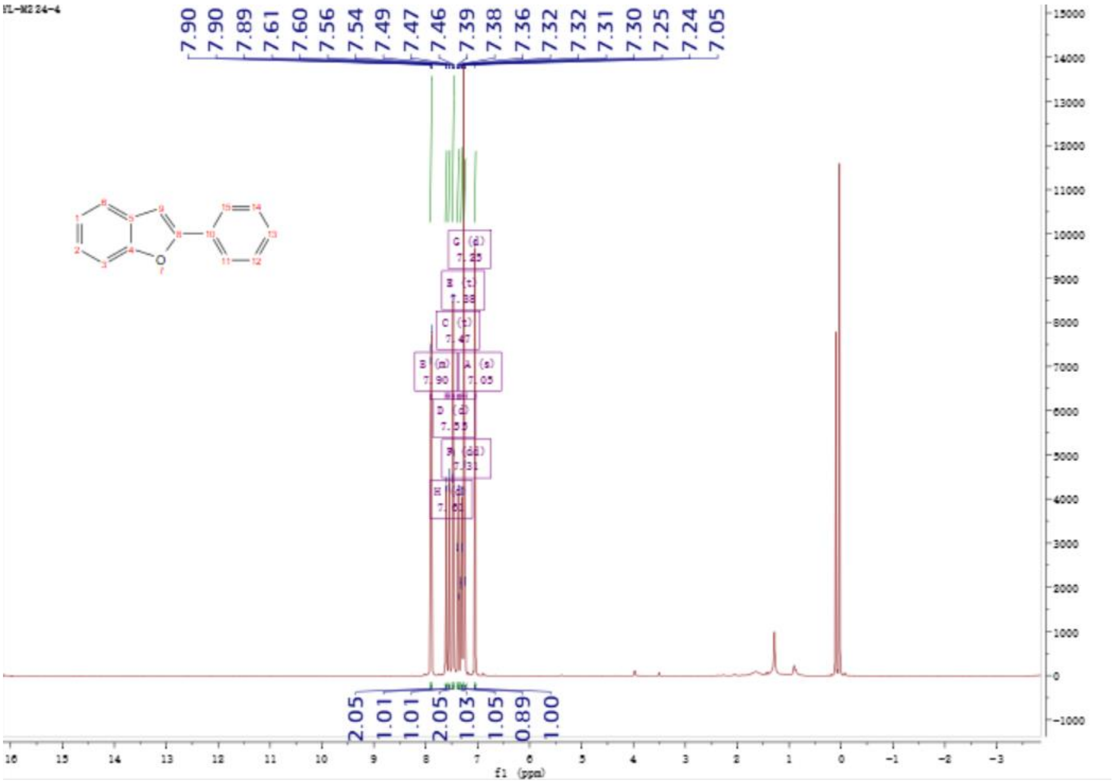

compound 1 ^{13}C NMR

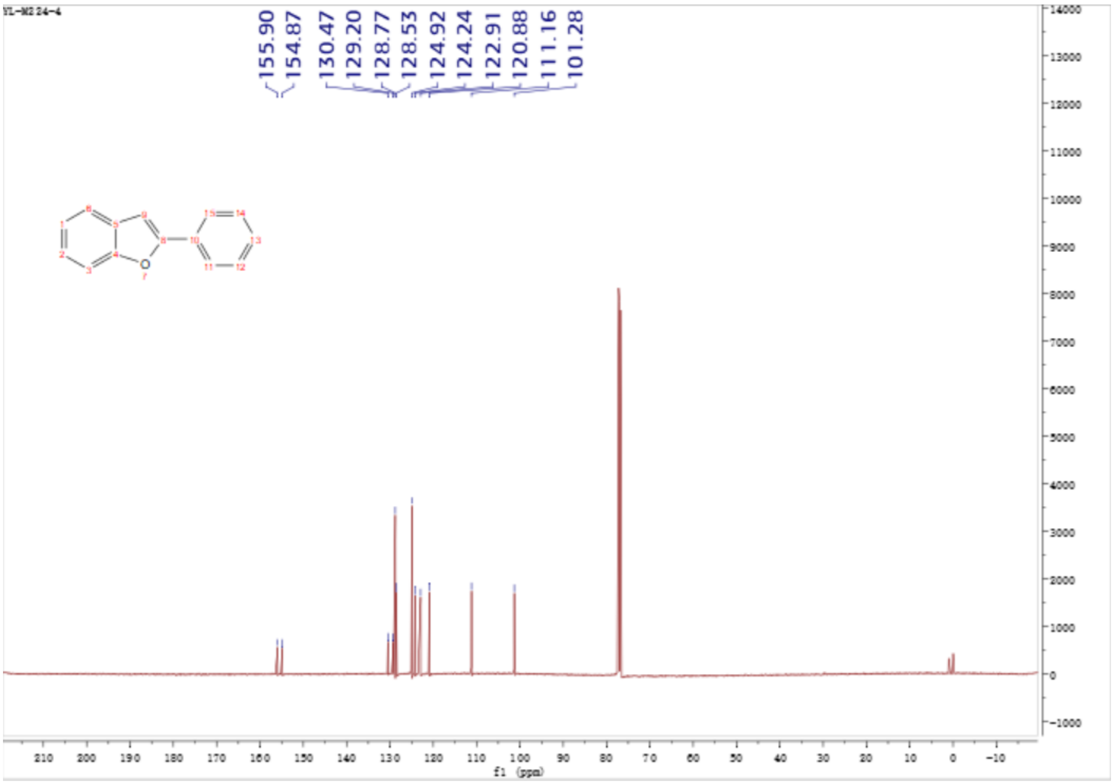

compound 2 ^{1}H NMR

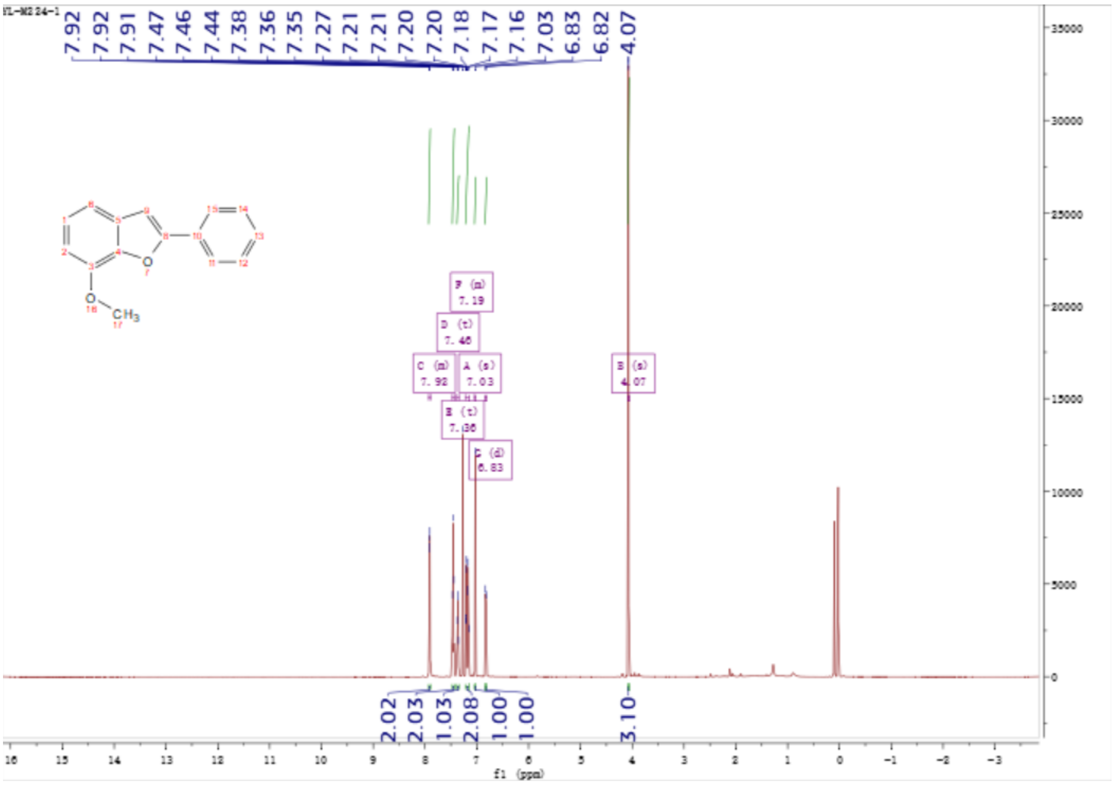

compound 2 ^{13}C NMR

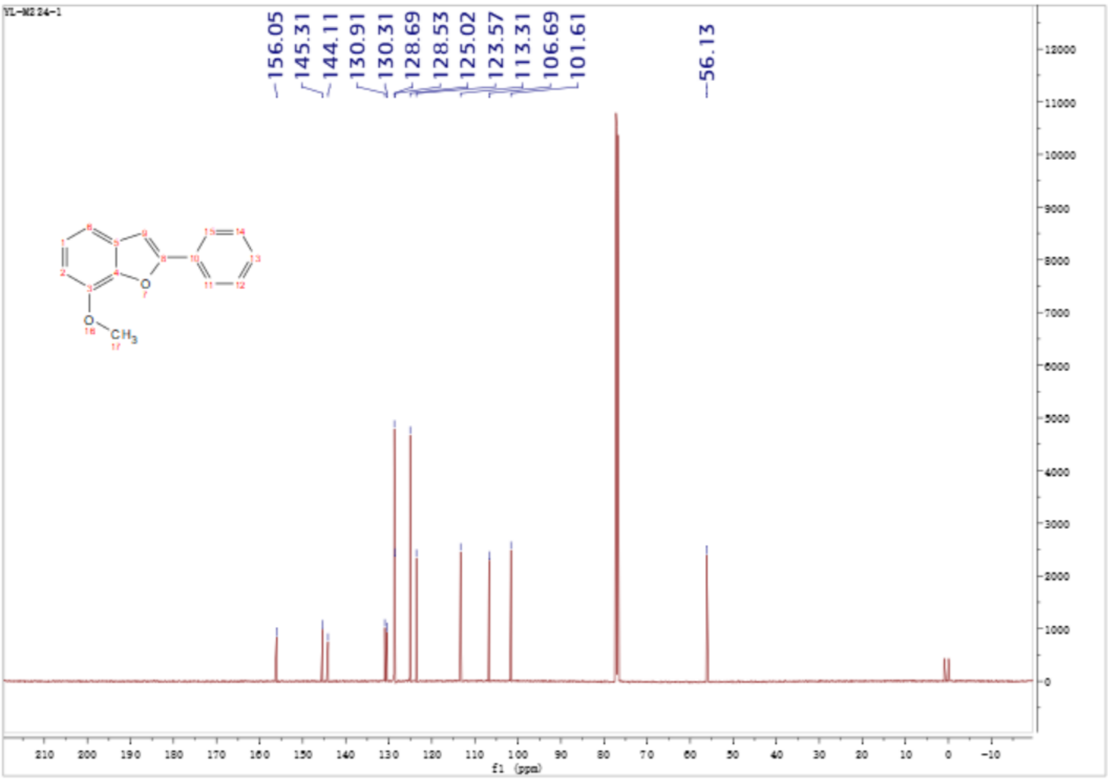

compound 3 ^{1}H NMR

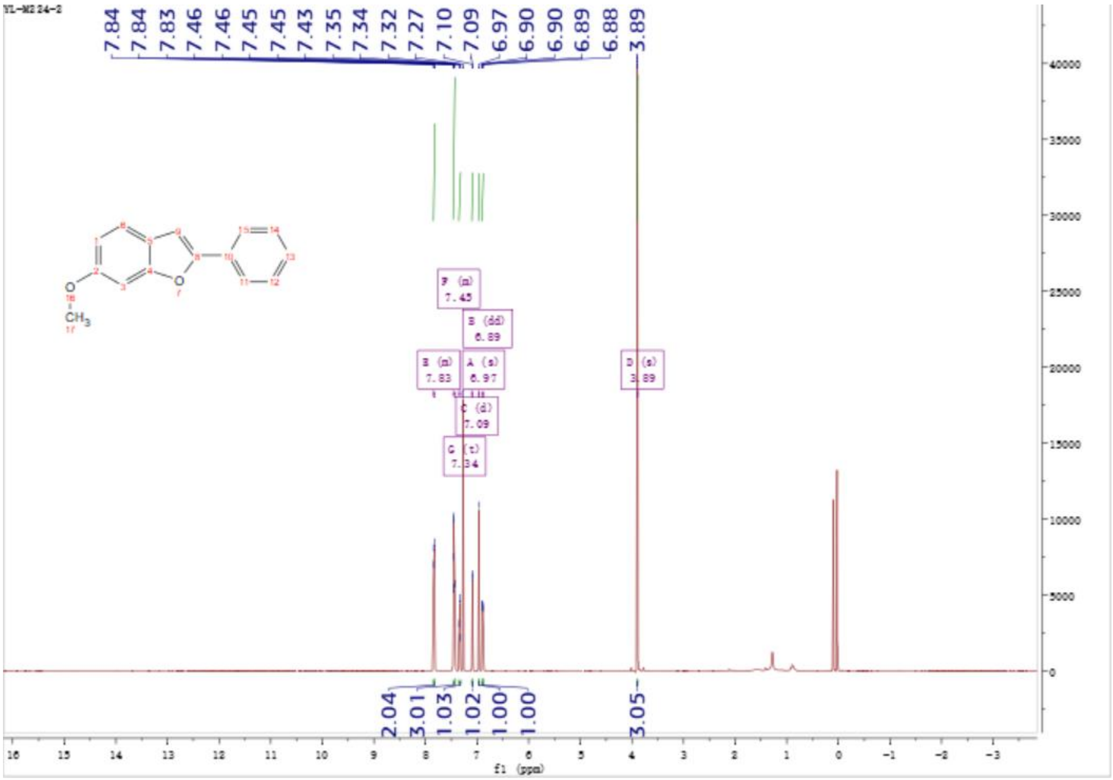

compound 3 ^{13}C NMR

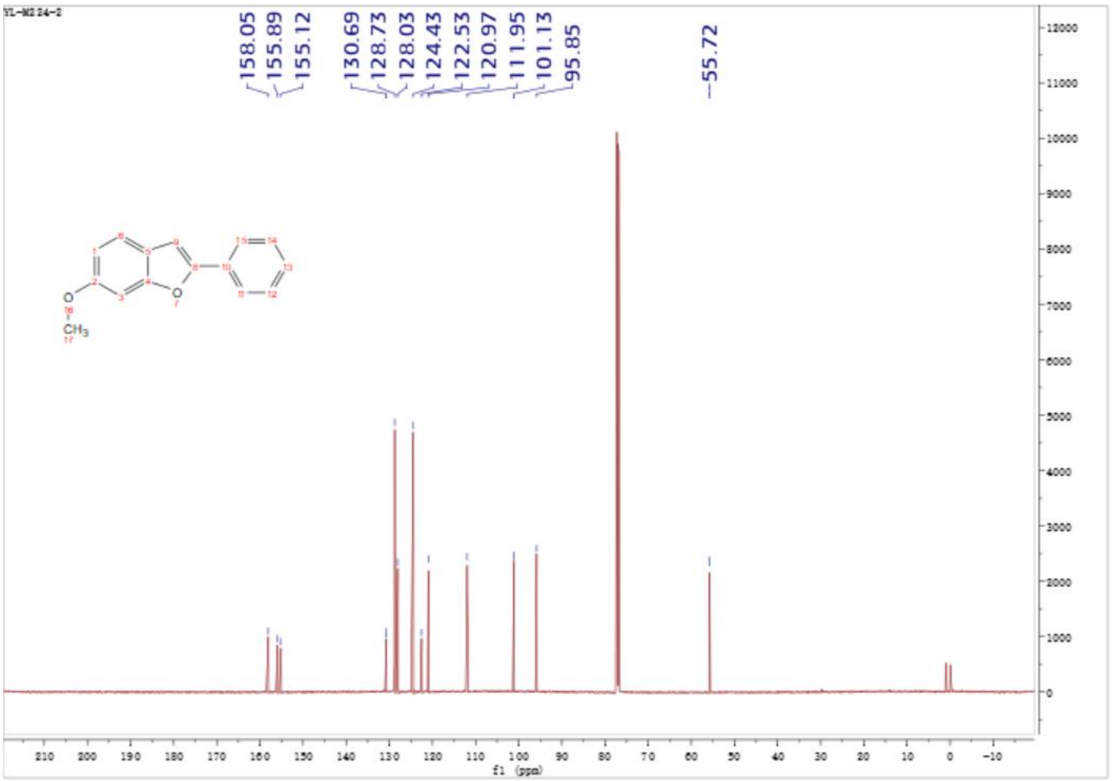

compound 4 ^{1}H NMR

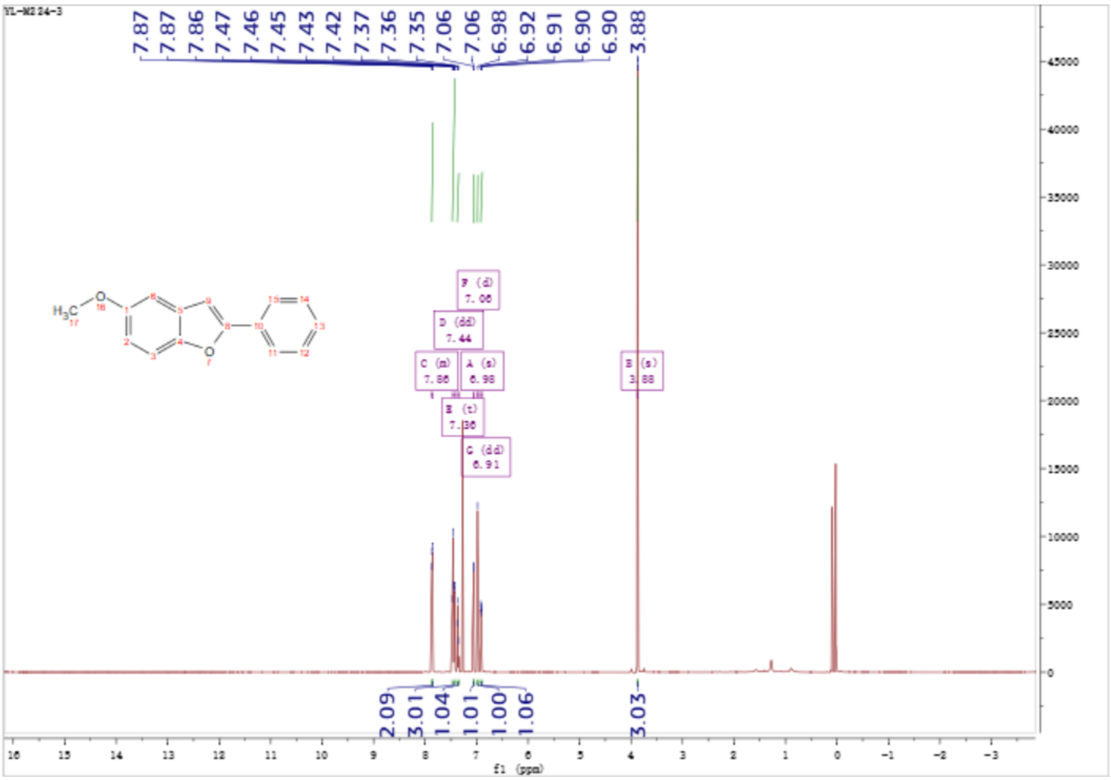

compound 4 ^{13}C NMR

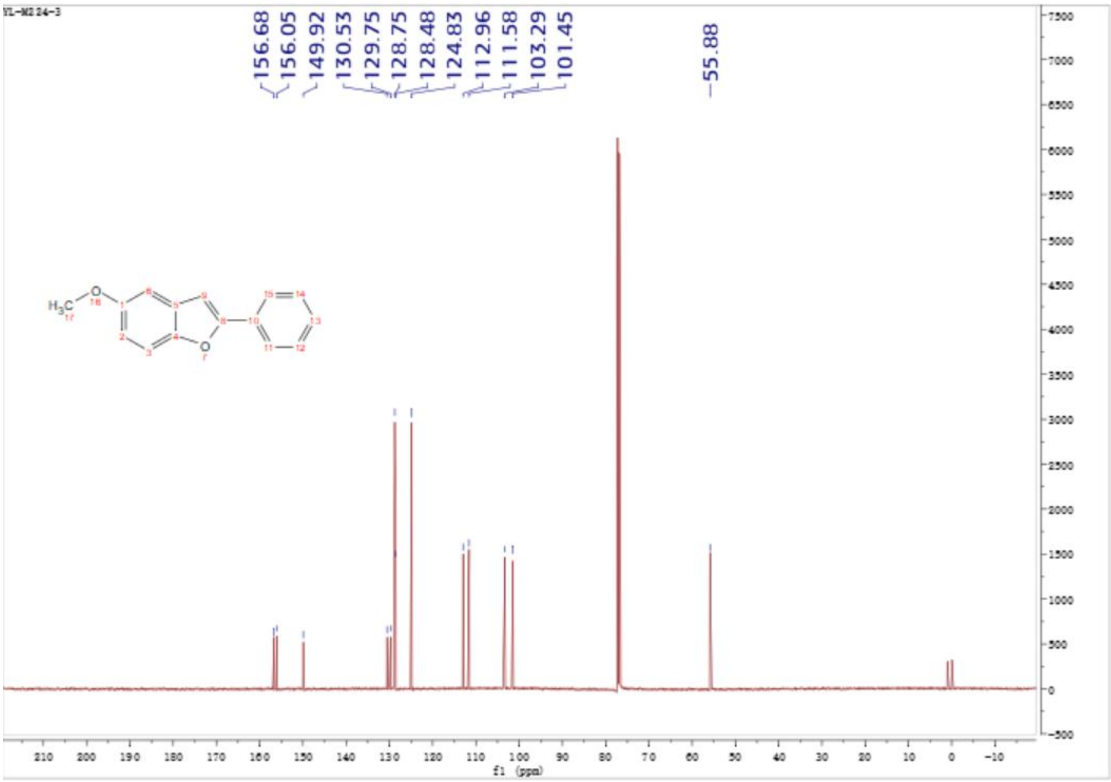

compound 5 ^{1}H NMR

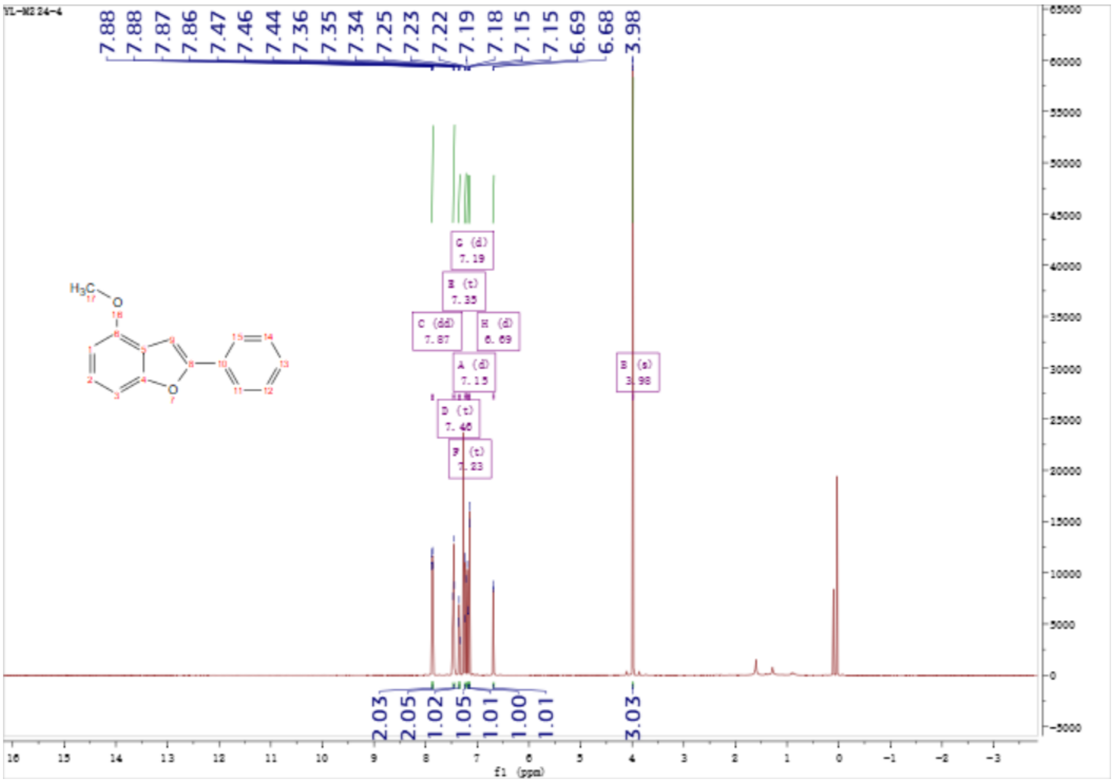

compound 5 ^{13}C NMR

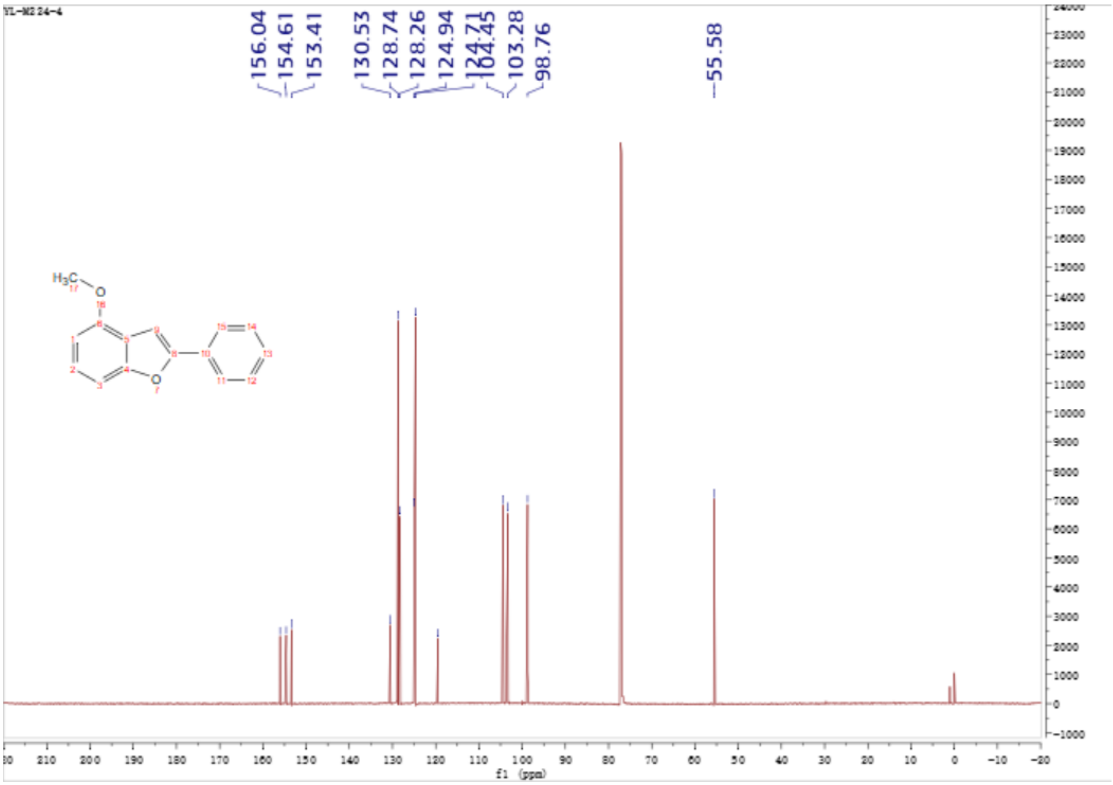

compound 6 ^{1}H NMR

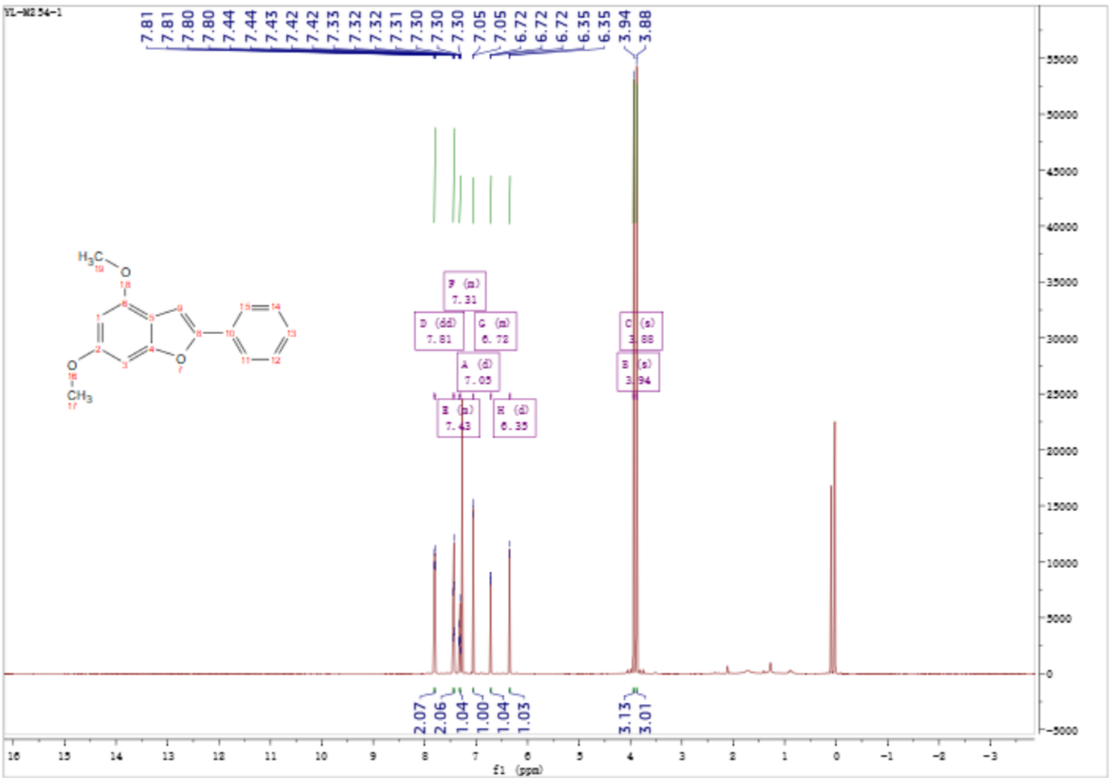

compound 6 ^{13}C NMR

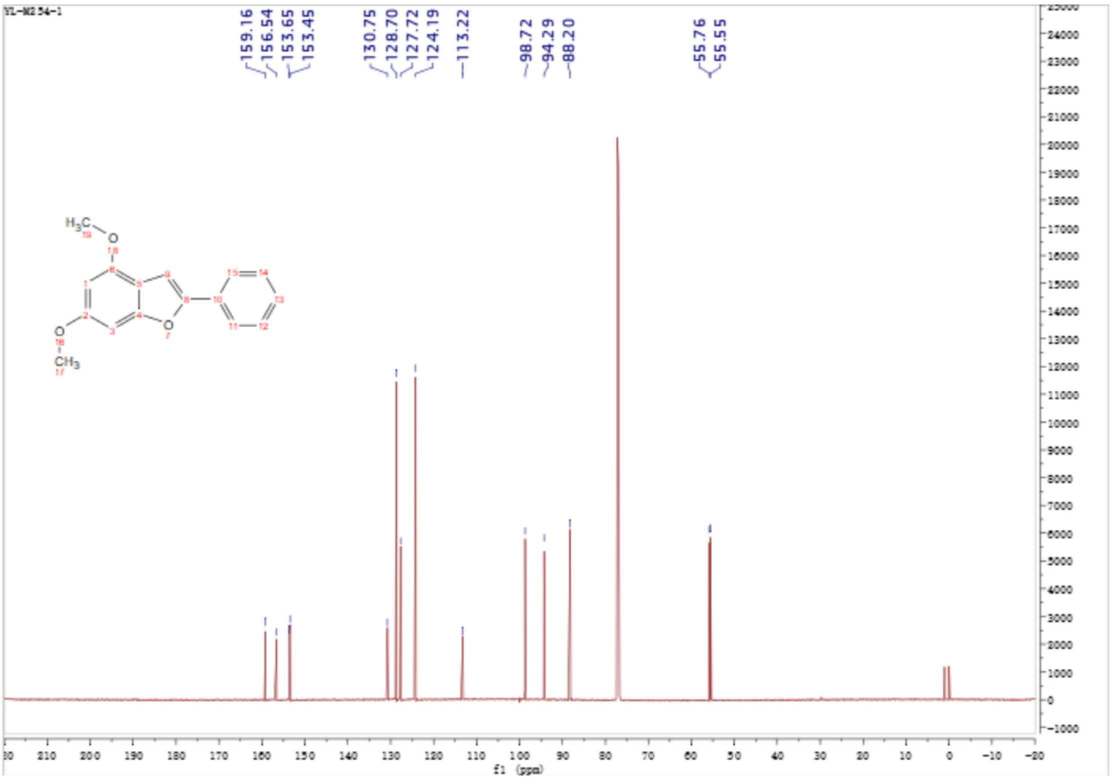

compound 7 ^{1}H NMR

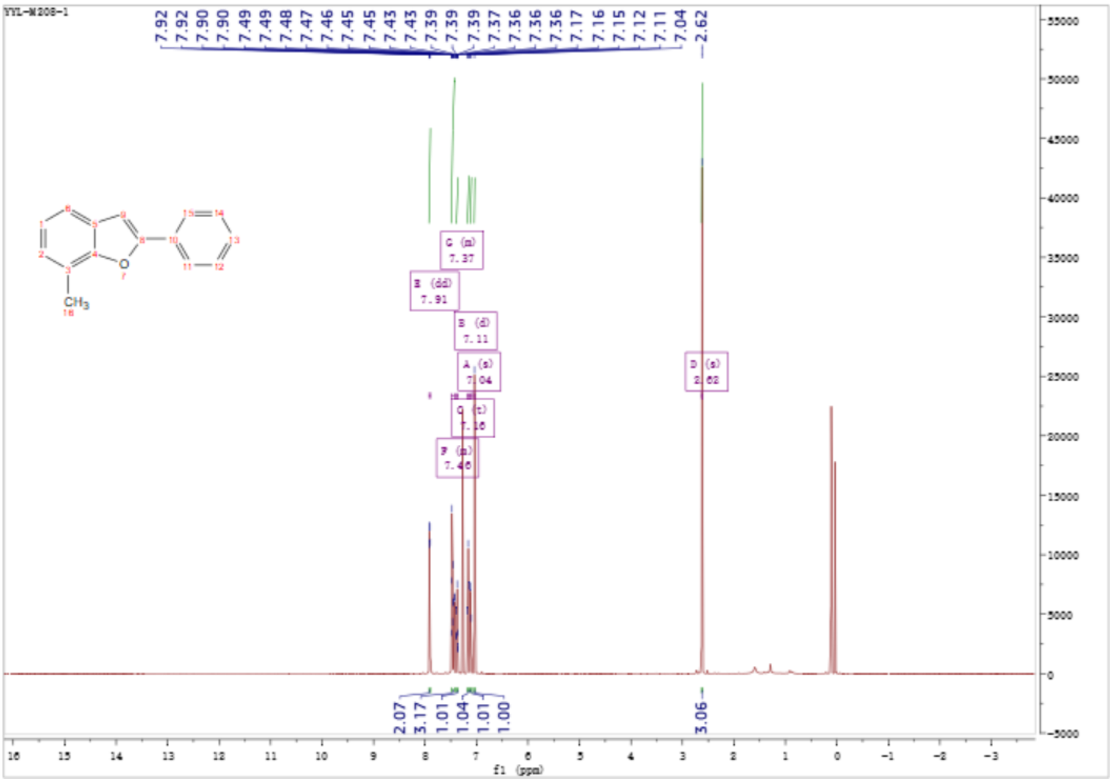

compound 7 ^{13}C NMR

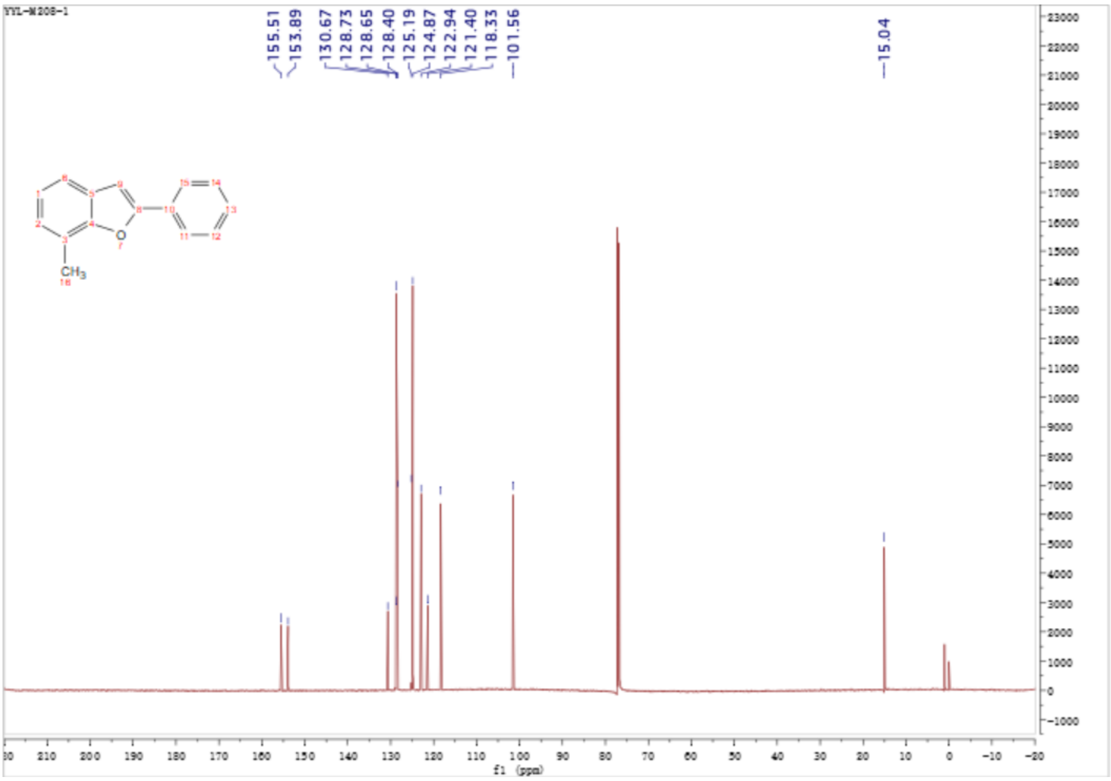

compound 8 ^{1}H NMR

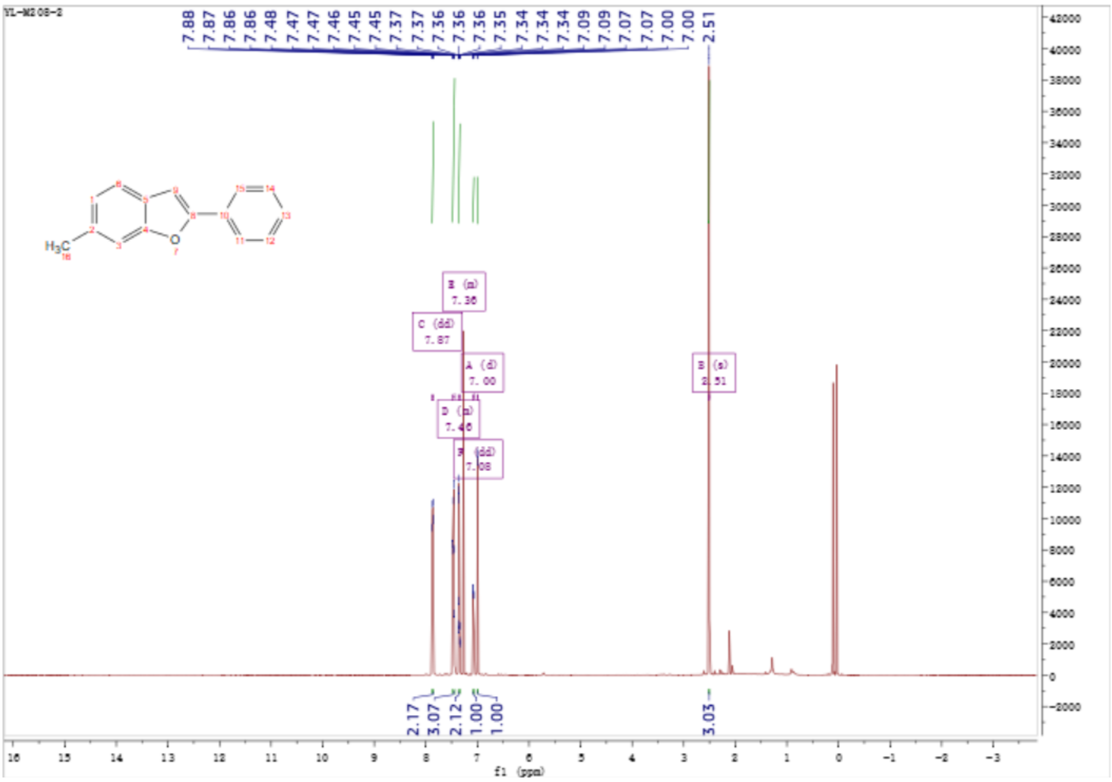

compound 8 ^{13}C NMR

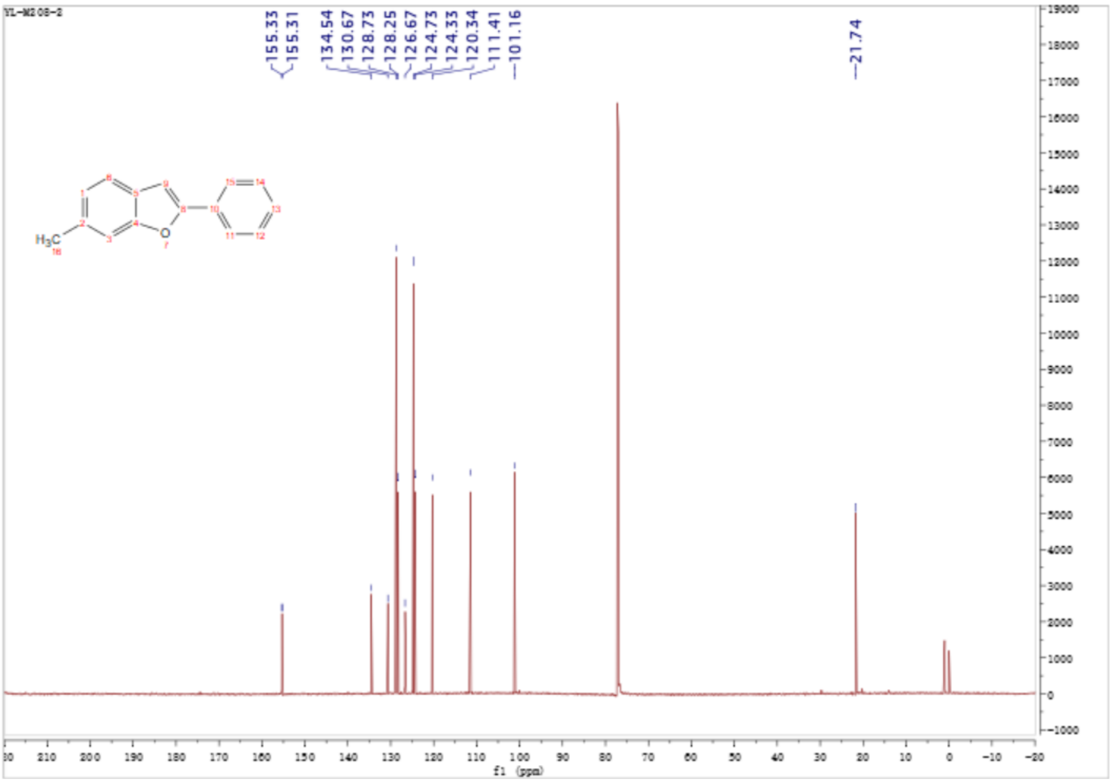

compound 9 ^{1}H NMR

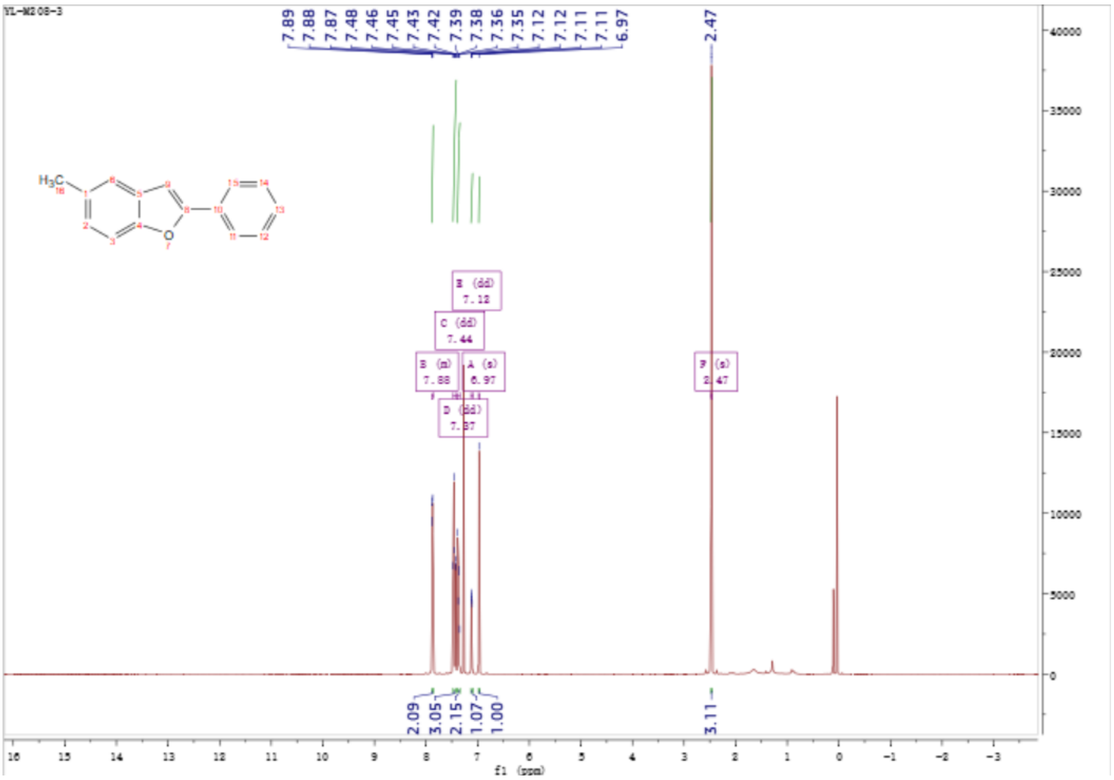

compound 9 ^{13}C NMR

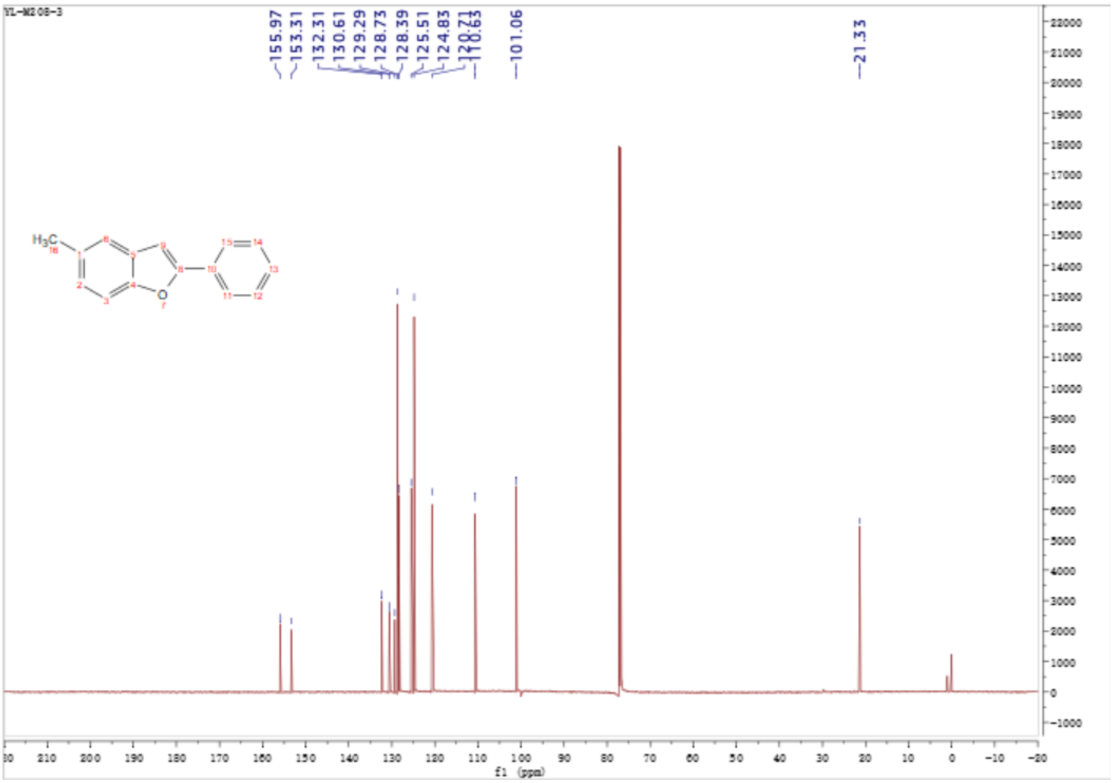

compound 10 ^{1}H NMR

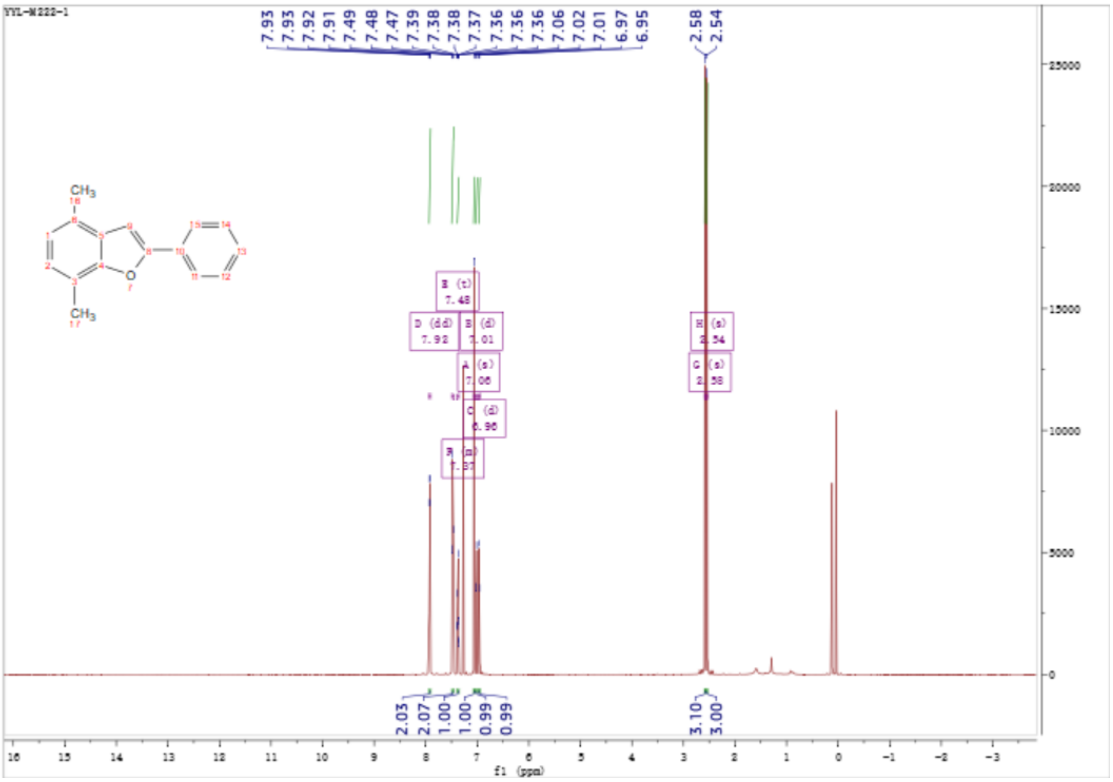

compound 10 ^{13}C NMR

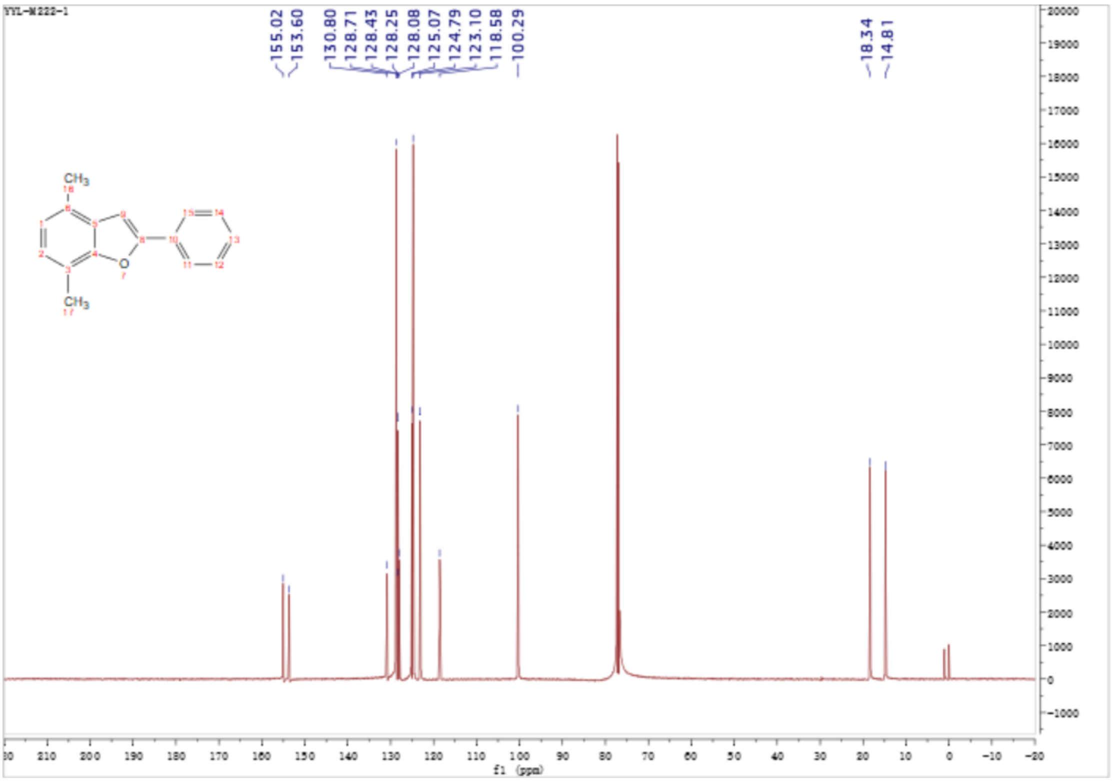

compound 11 ^{1}H NMR

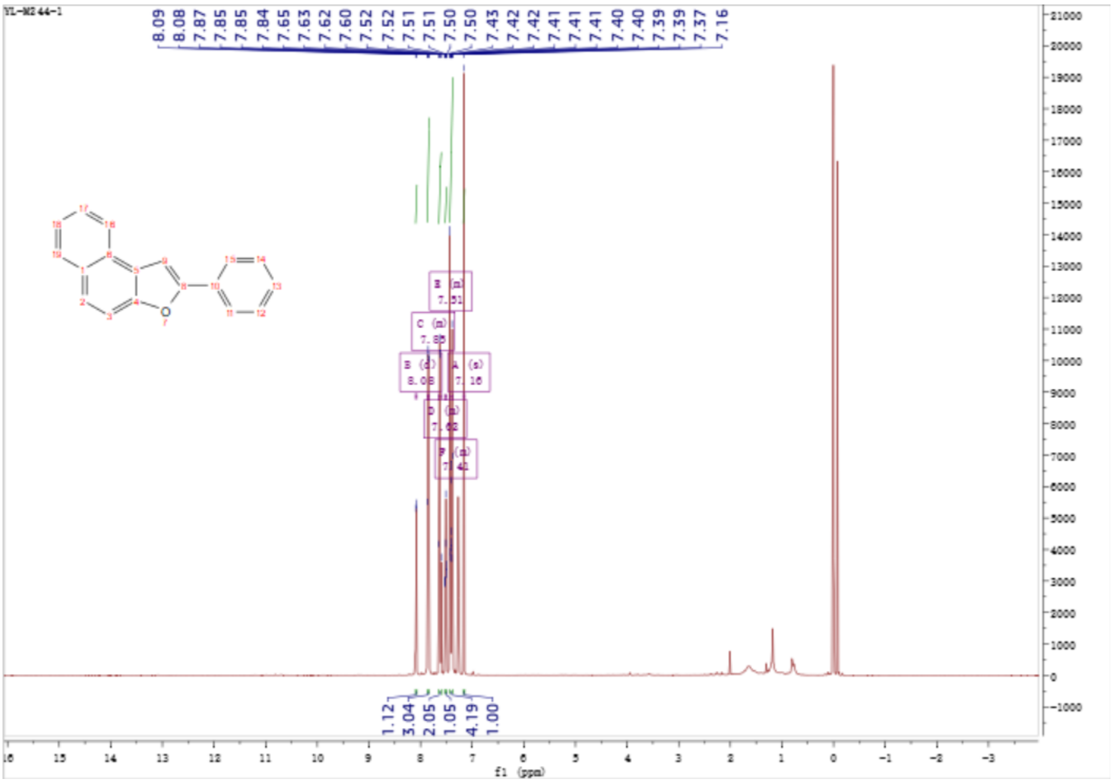

compound 11 ^{13}C NMR

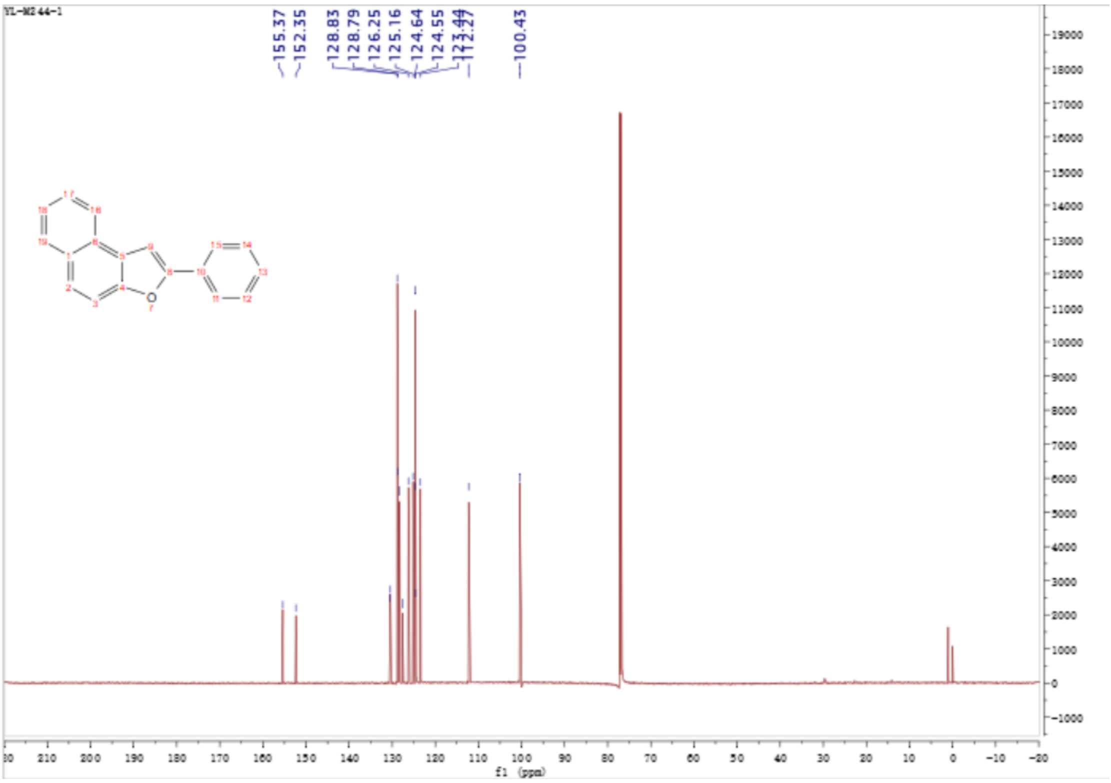

compound 12 ^{1}H NMR

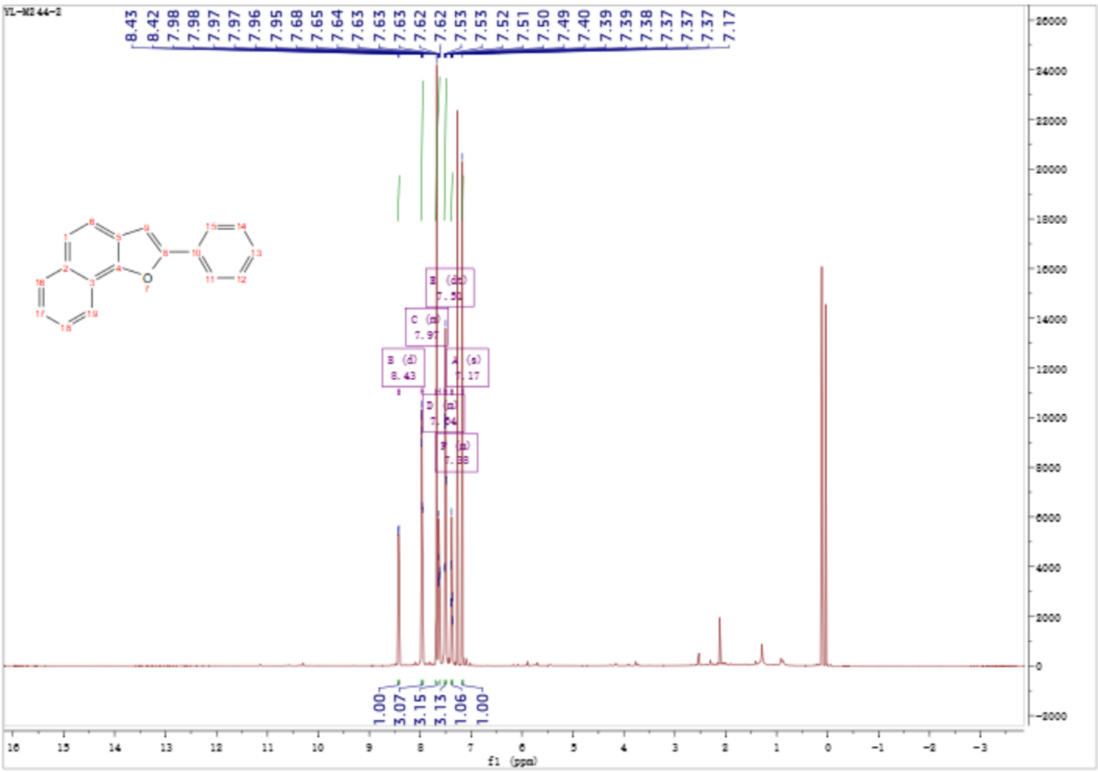

compound 12 ^{13}C NMR

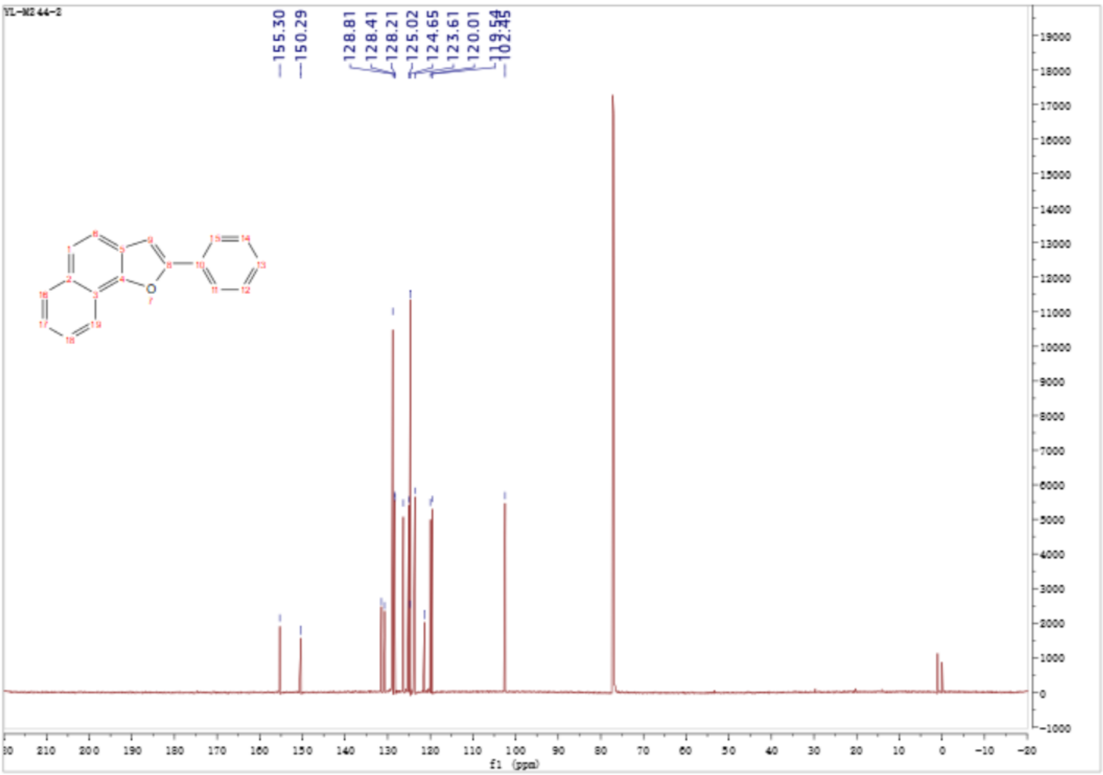

compound 13 ^{1}H NMR

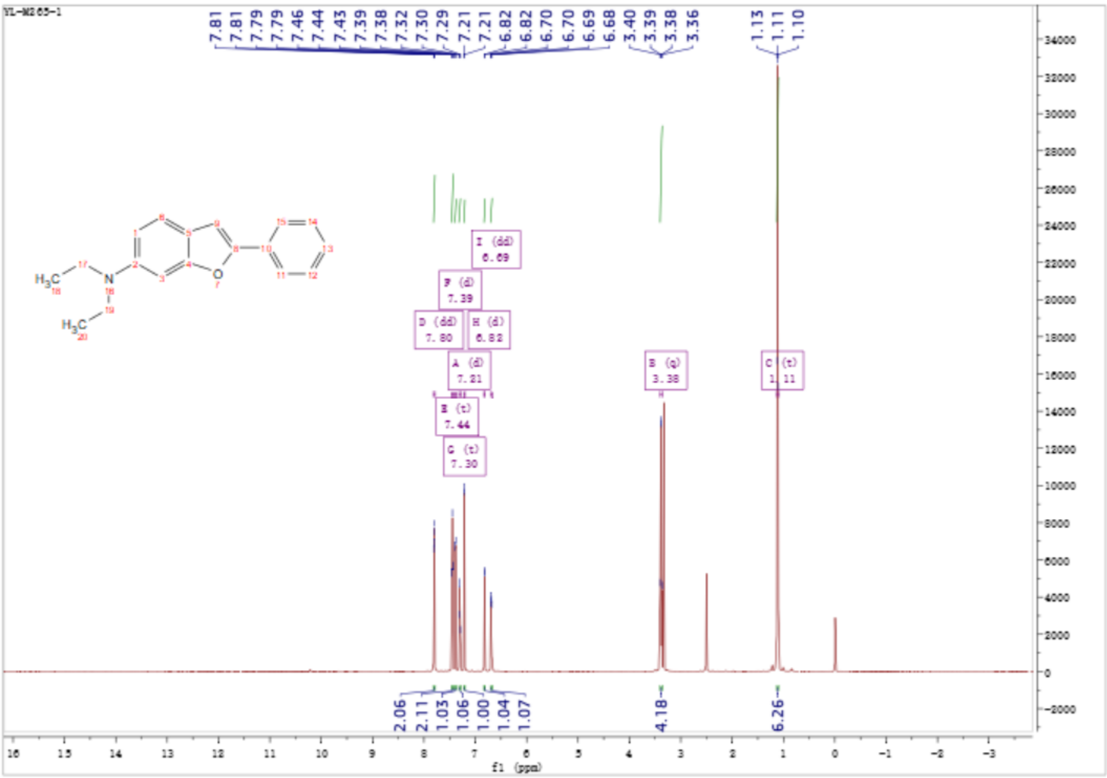

compound 13 ^{13}C NMR

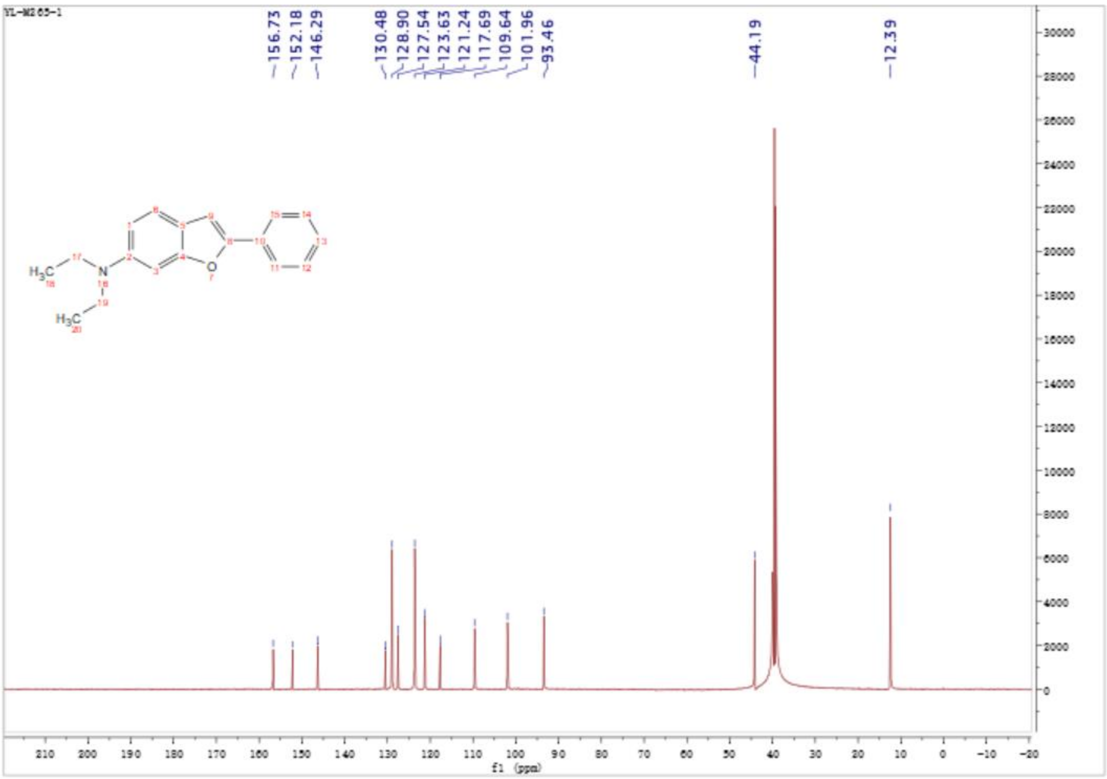

compound 14 ^{1}H NMR

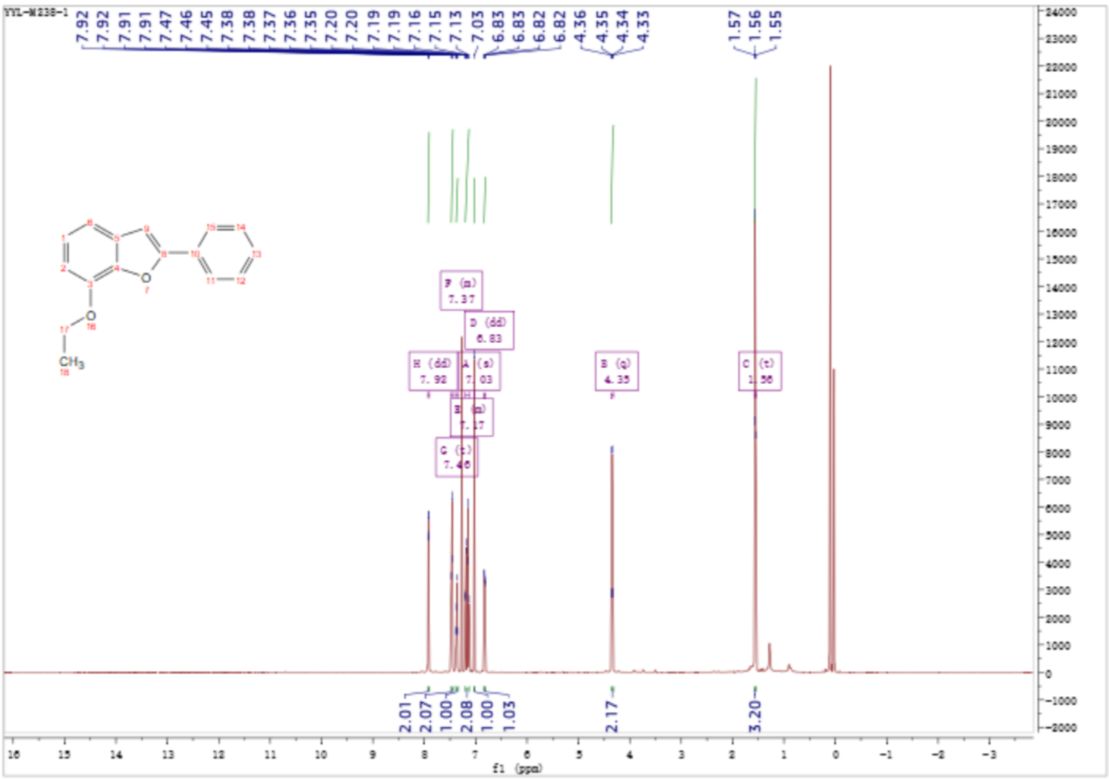

compound 14 ^{13}C NMR

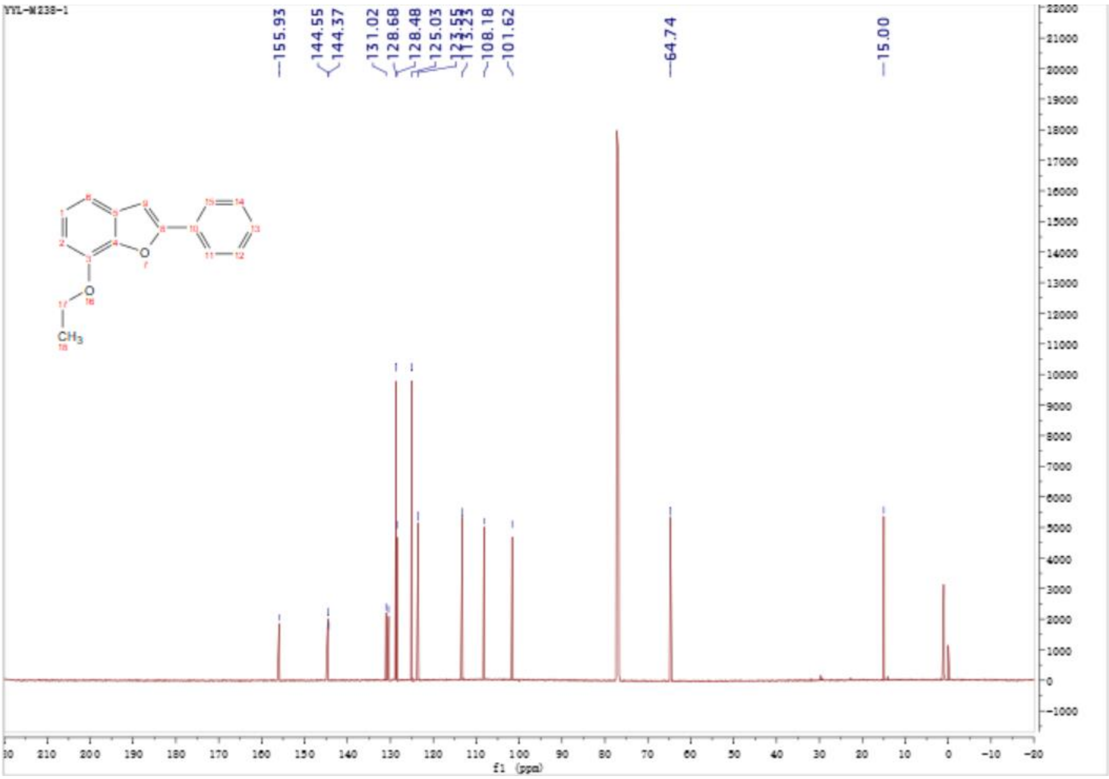

compound 15 ^{1}H NMR

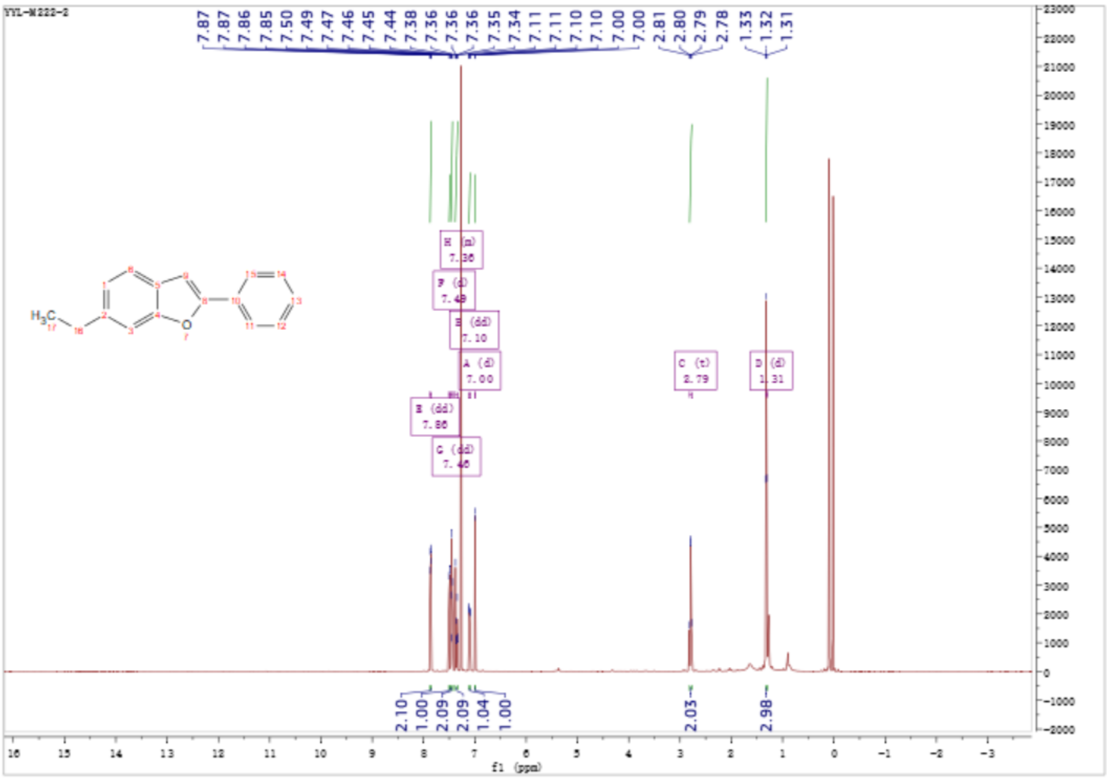

compound 15 ^{13}C NMR

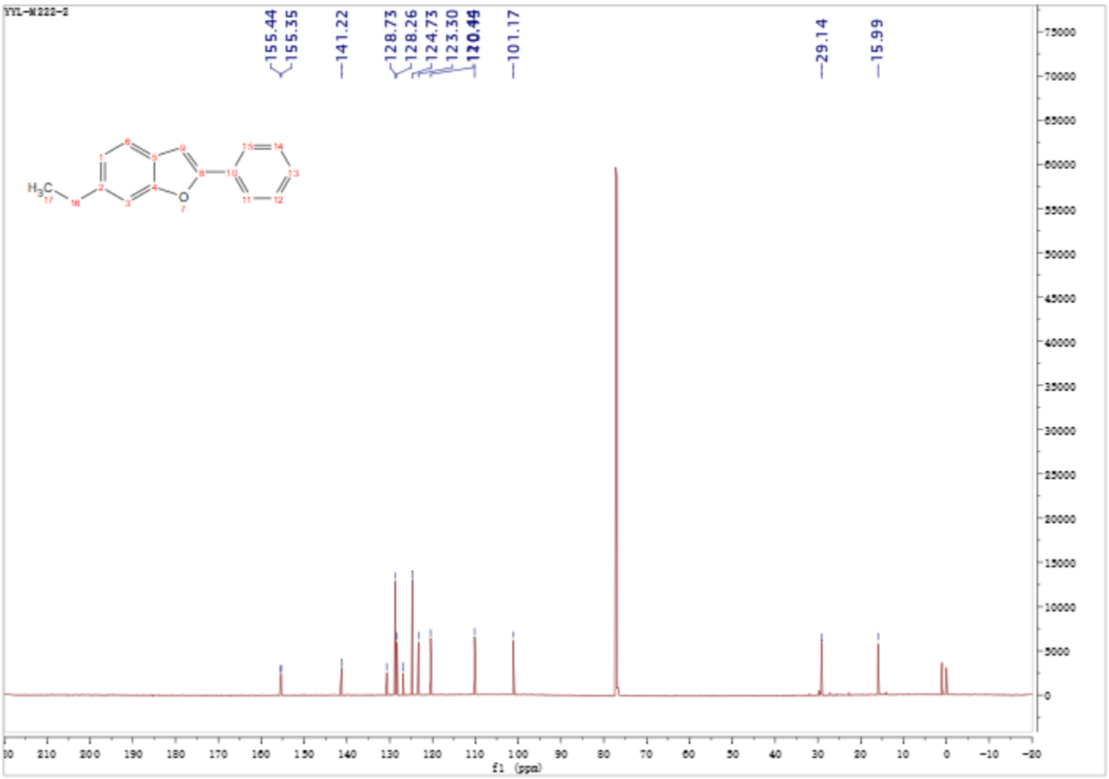

compound 16 ^{1}H NMR

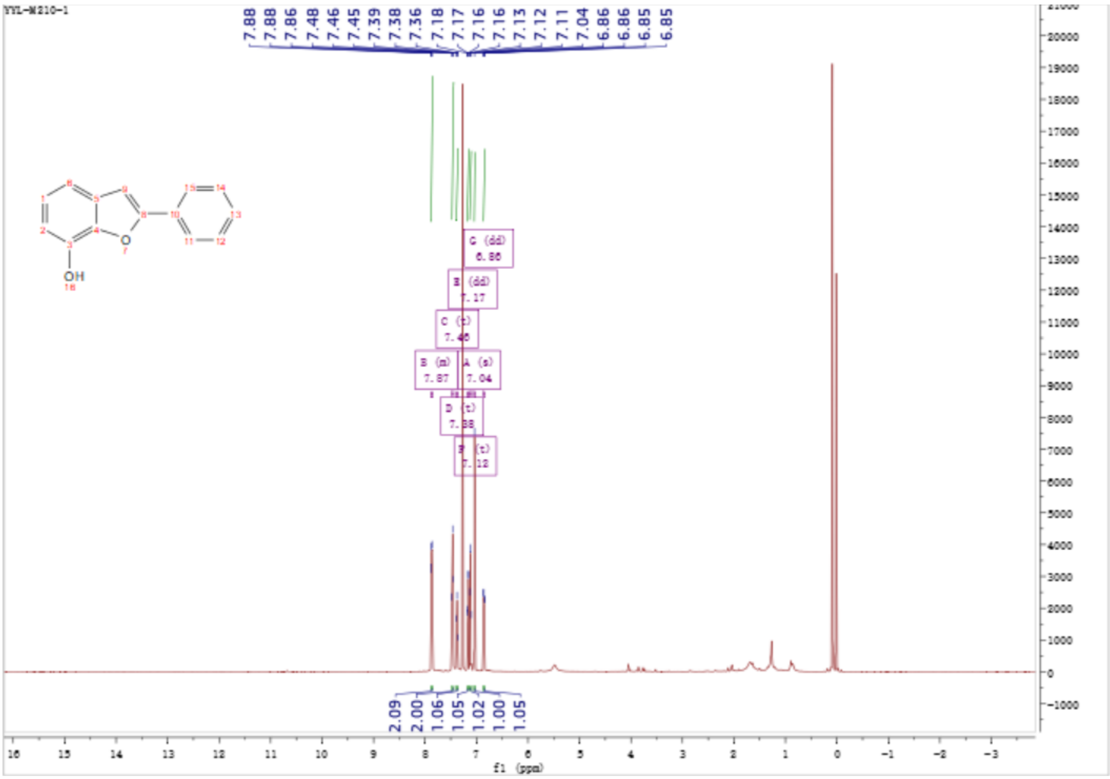

compound 16 ^{13}C NMR

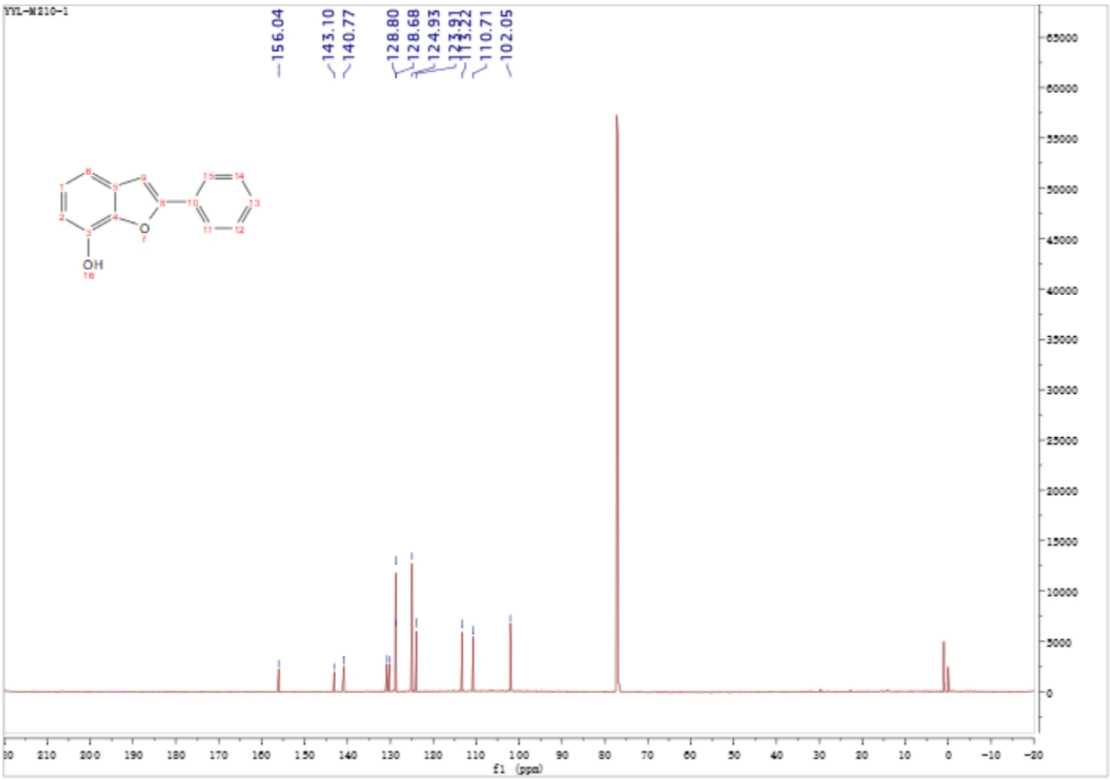

compound 17 ^{1}H NMR

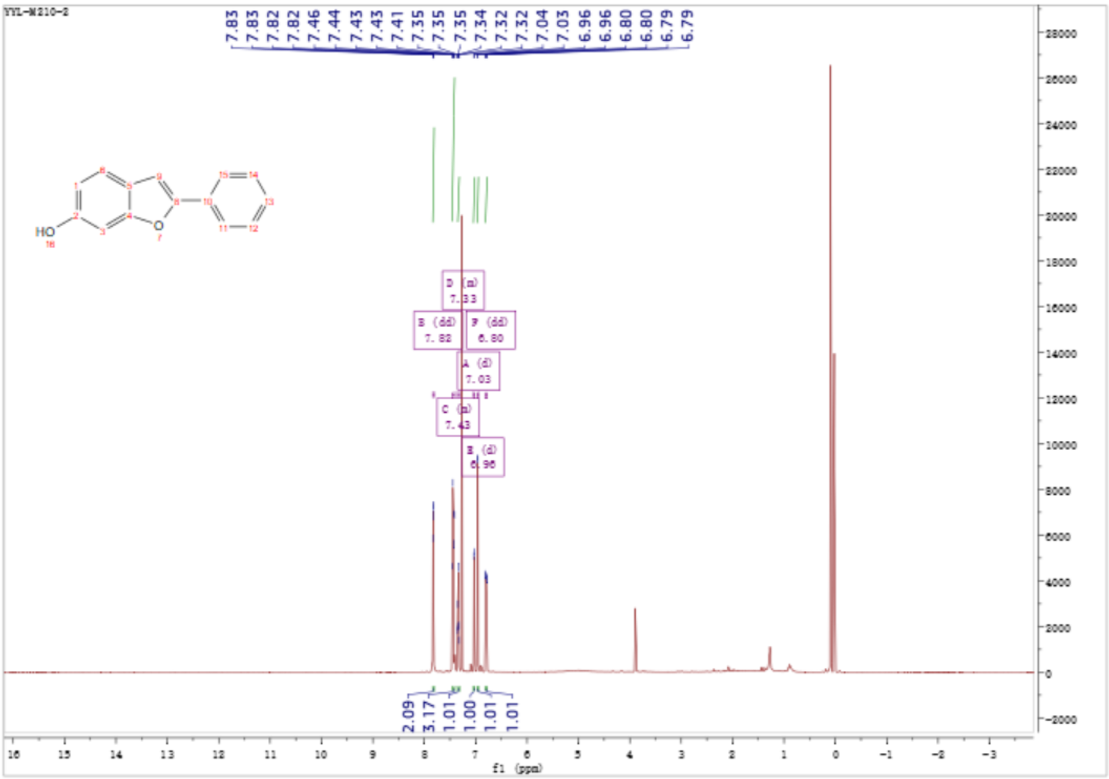

compound 17 ^{13}C NMR

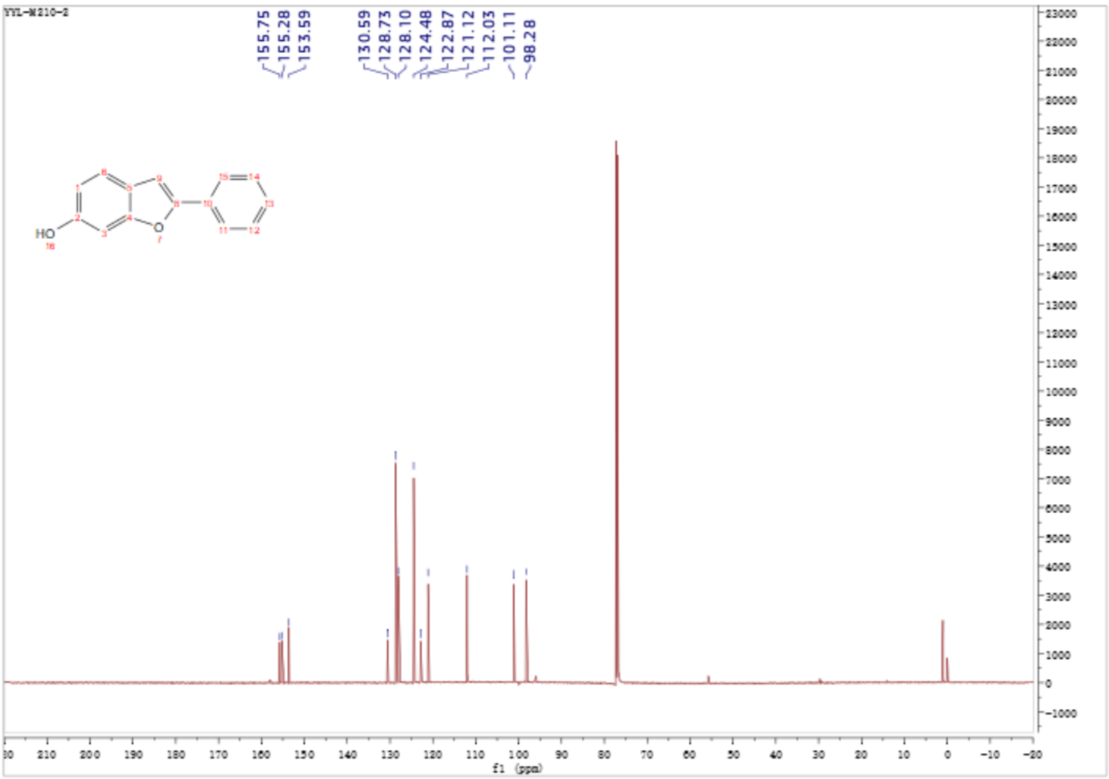

compound 18 ^{1}H NMR

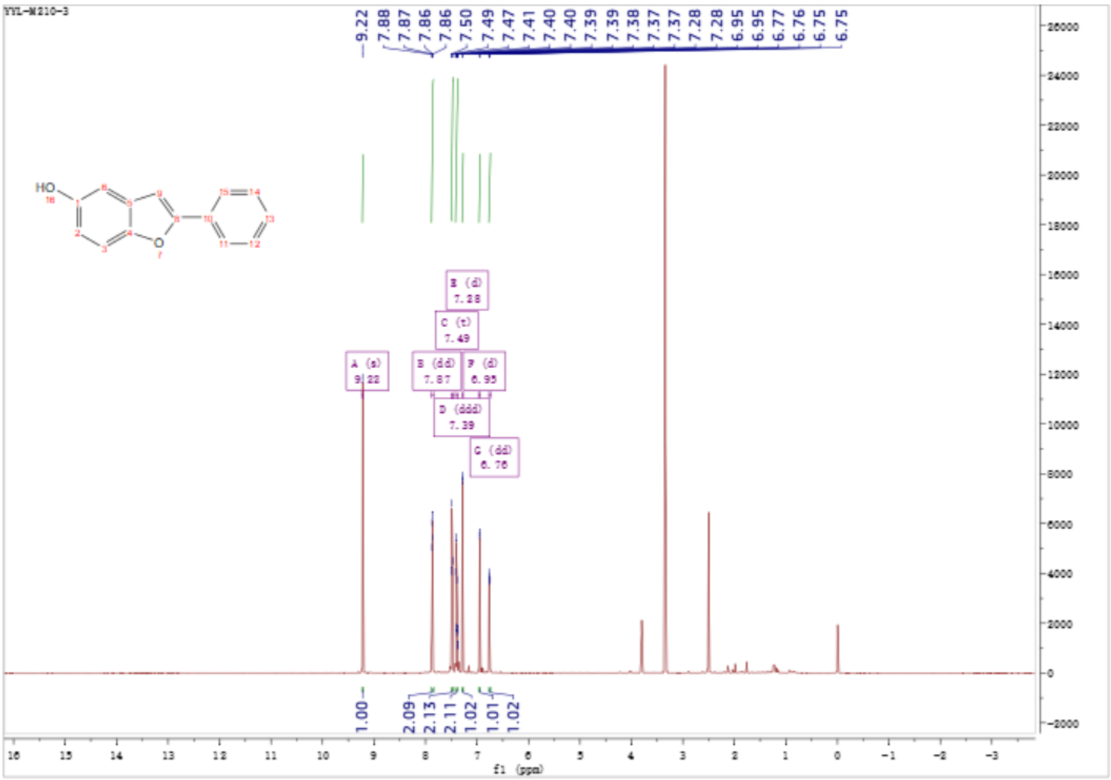

compound 18 ^{13}C NMR

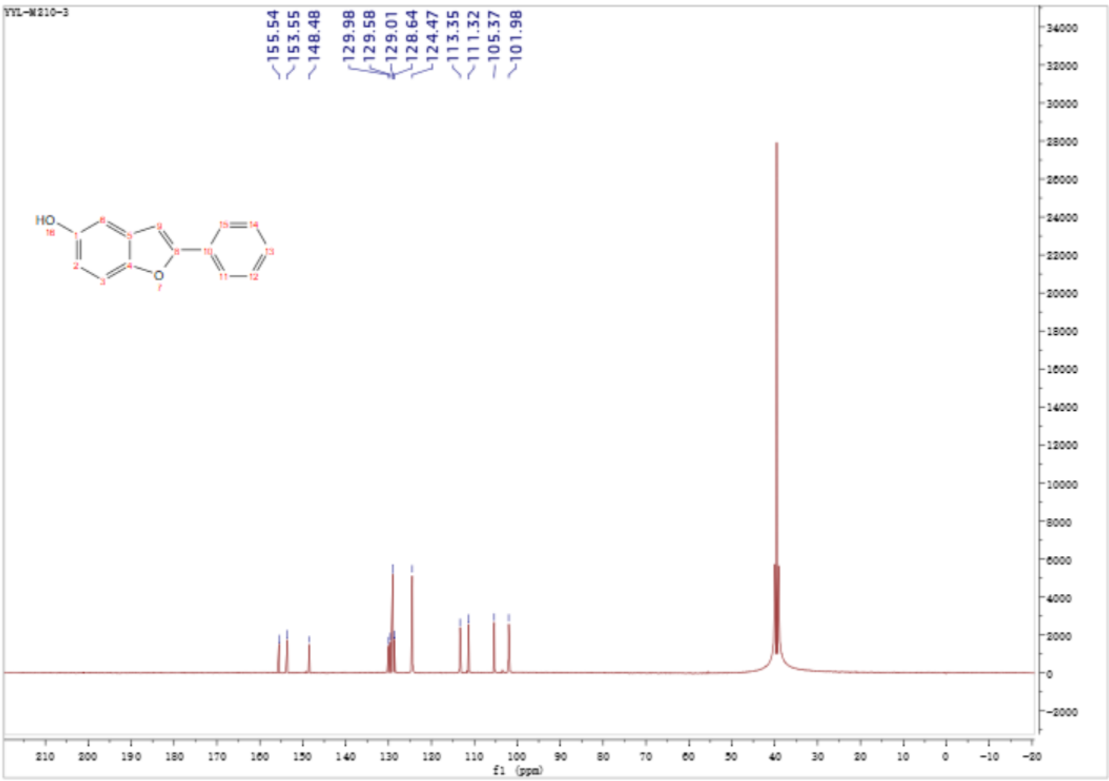

compound 19 ^{1}H NMR

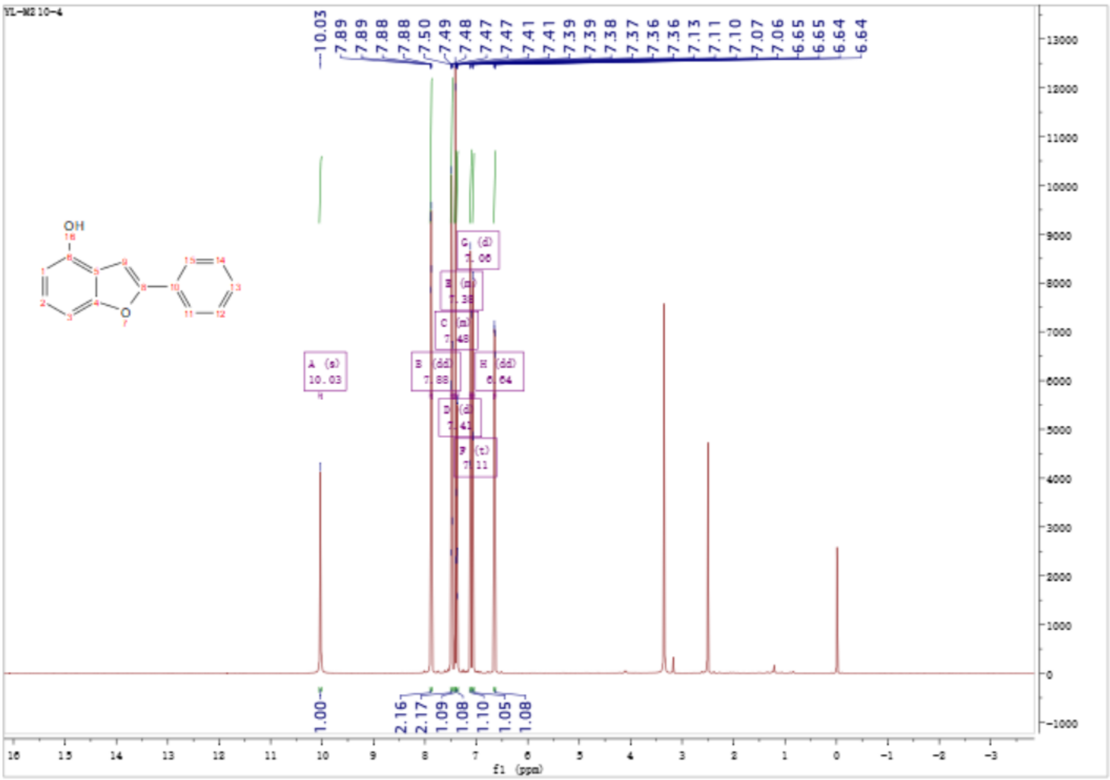

compound 19 ^{13}C NMR

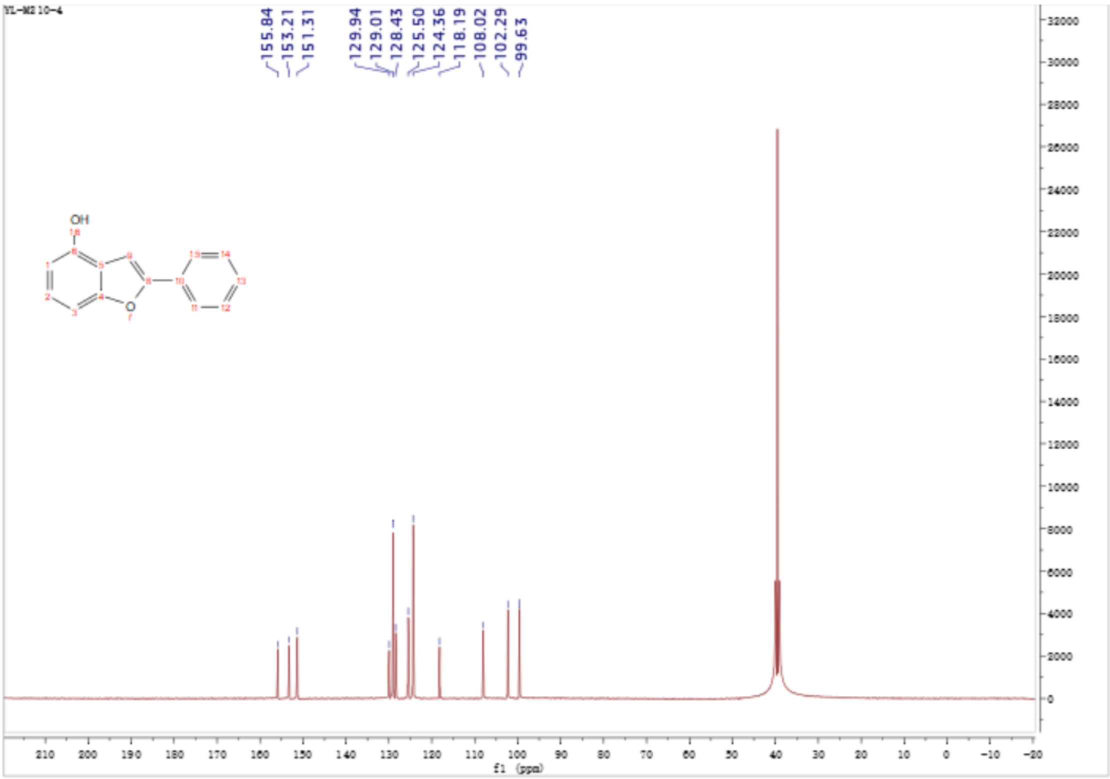

compound 20 ^{1}H NMR

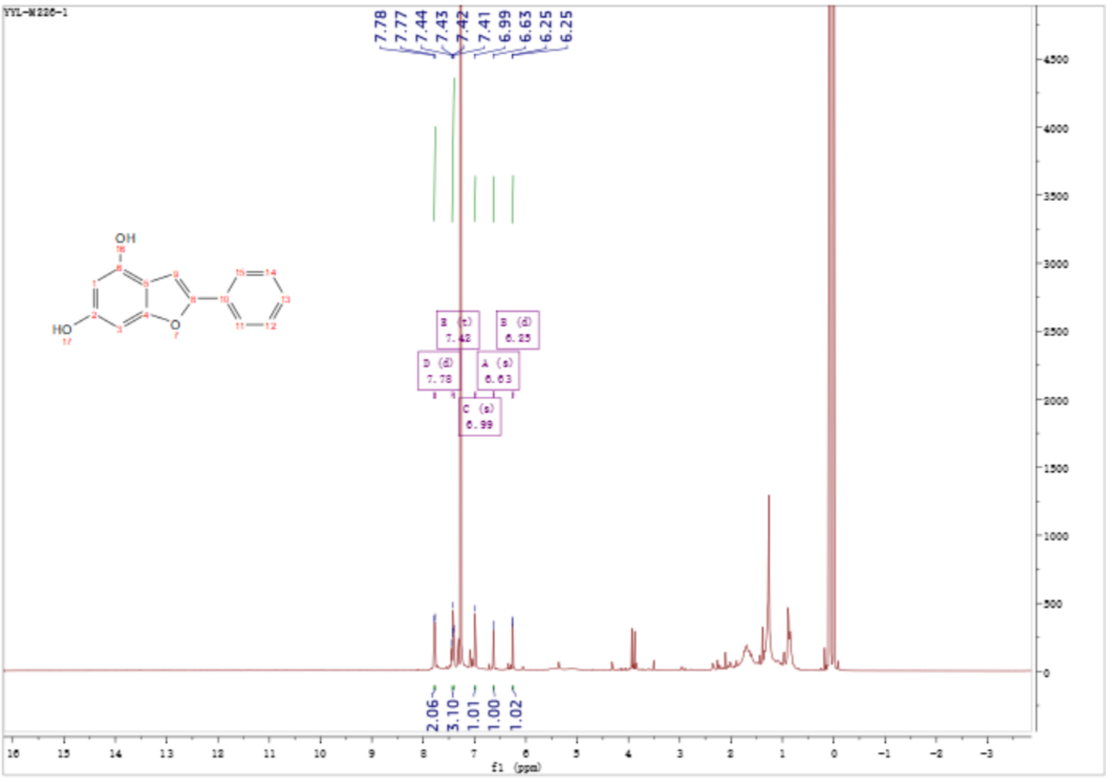

compound 20 ^{13}C NMR

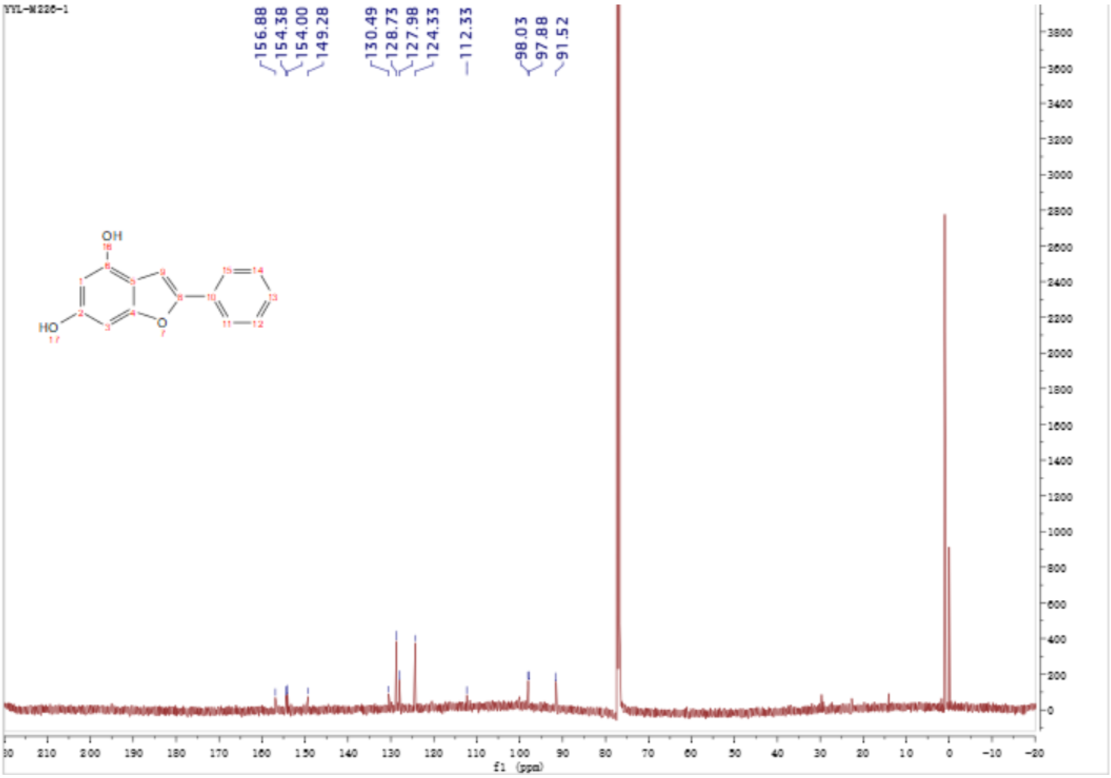

compound 21 ^{1}H NMR

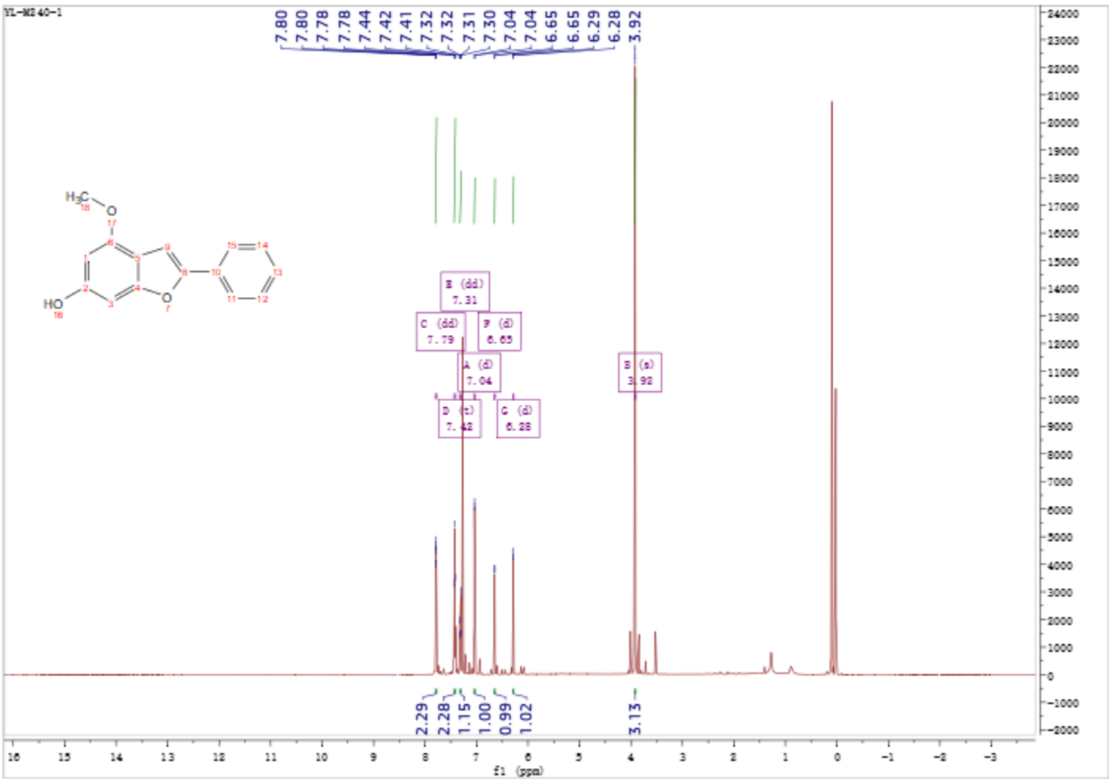

compound 21 ^{13}C NMR

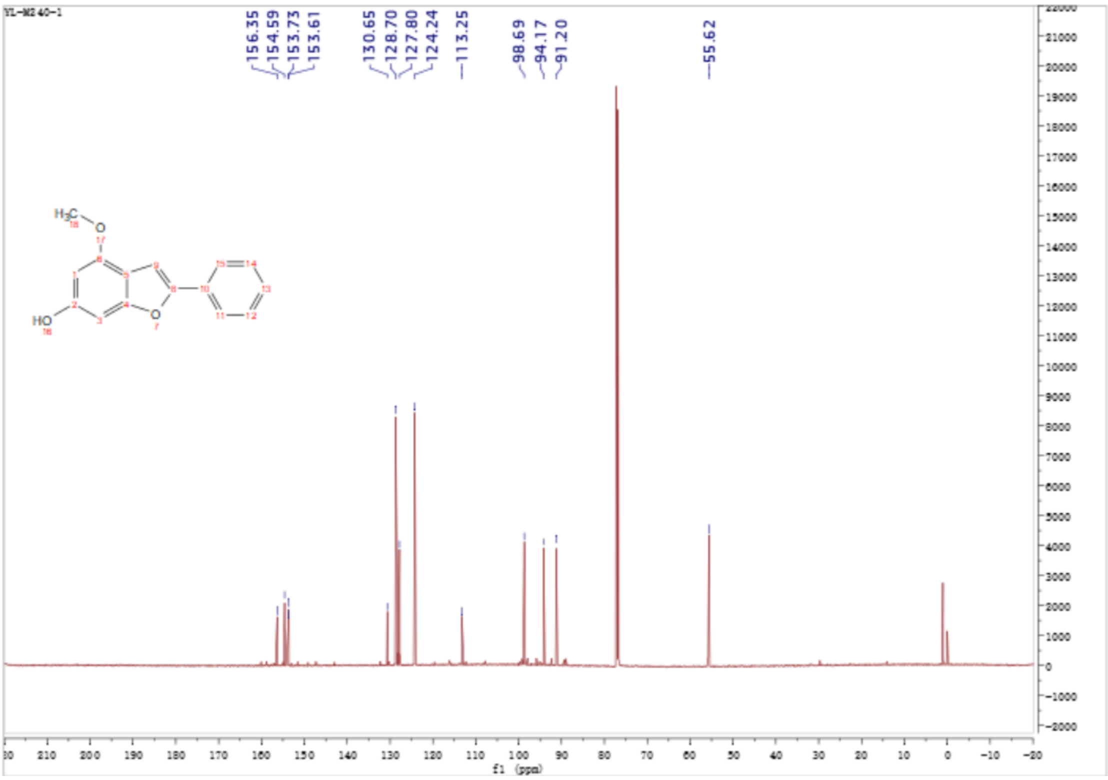

compound 22 ^{1}H NMR

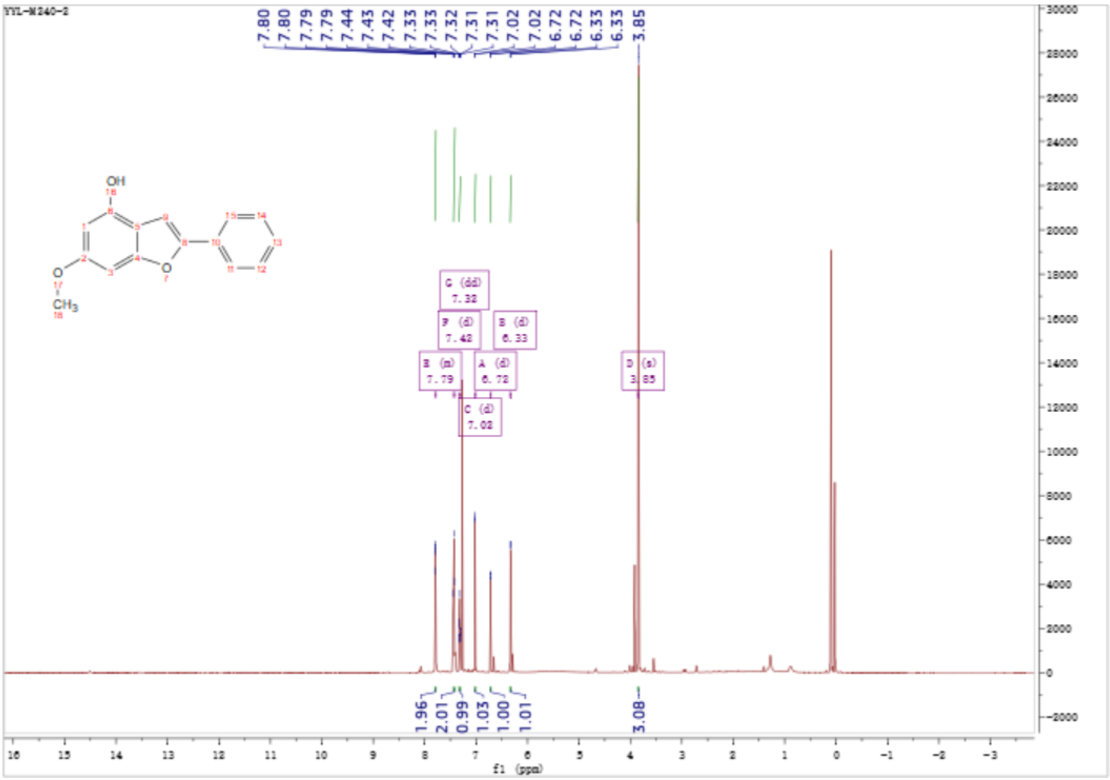

compound 22 ^{13}C NMR

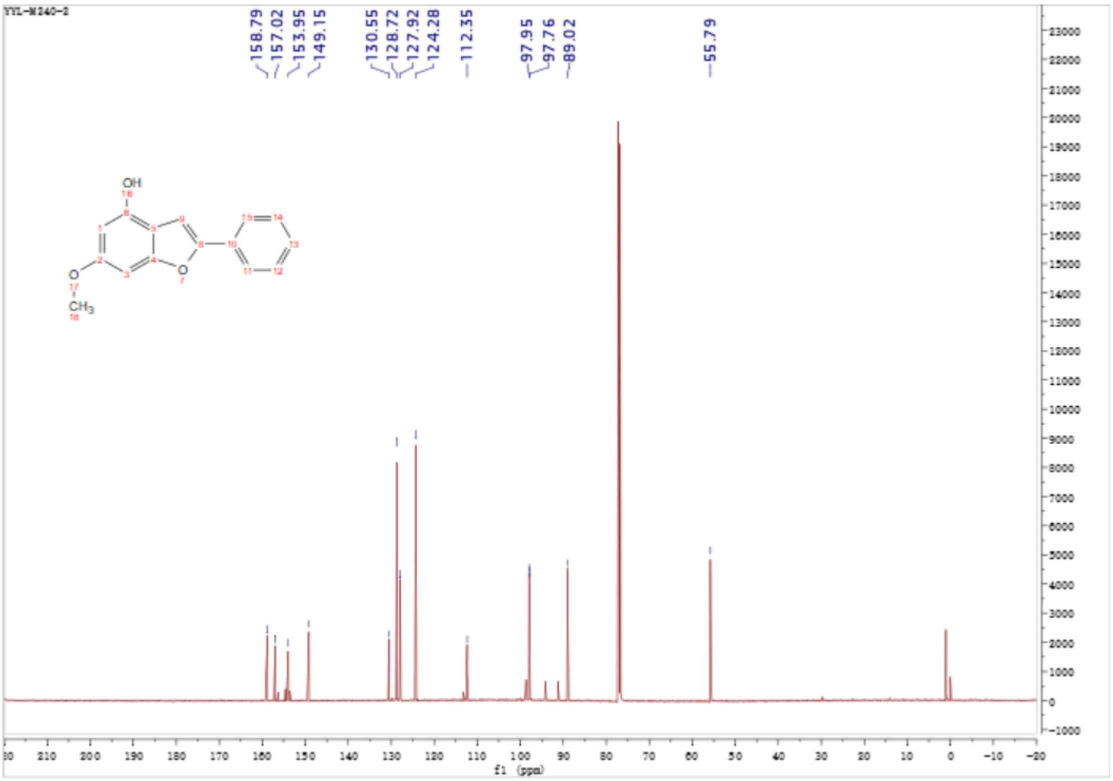

Supplement: Supplemental Material [file IENZ_A_1940993_SM8199.pdf]
